# The murine IgH locus contains a distinct DNA sequence motif for the chromatin regulatory factor CTCF

**DOI:** 10.1074/jbc.RA118.007348

**Published:** 2019-07-08

**Authors:** David N. Ciccone, Yuka Namiki, Changfeng Chen, Katrina B. Morshead, Andrew L. Wood, Colette M. Johnston, John W. Morris, Yanqun Wang, Ruslan Sadreyev, Anne E. Corcoran, Adam G. W. Matthews, Marjorie A. Oettinger

**Affiliations:** ‡ Department of Molecular Biology, Massachusetts General Hospital, and Department of Genetics, Harvard Medical School, Boston, Massachusetts 02114; § Lymphocyte Signalling and Development, Babraham Institute, Babraham Research Campus, Cambridge CB22 3AT, United Kingdom; ¶ Department of Biological Sciences and Program in Biochemistry, Wellesley College, Wellesley, Massachusetts 02481

**Keywords:** DNA recombination, chromatin, cellular immune response, chromatin immunoprecipitation (ChIP), chromatin regulation, adaptive immunity, antigen receptor, CCTC-binding factor (CTCF), DNA binding, V(D)J

## Abstract

Antigen receptor assembly in lymphocytes involves stringently-regulated coordination of specific DNA rearrangement events across several large chromosomal domains. Previous studies indicate that transcription factors such as paired box 5 (PAX5), Yin Yang 1 (YY1), and CCCTC-binding factor (CTCF) play a role in regulating the accessibility of the antigen receptor loci to the V(D)J recombinase, which is required for these rearrangements. To gain clues about the role of CTCF binding at the murine immunoglobulin heavy chain (IgH) locus, we utilized a computational approach that identified 144 putative CTCF-binding sites within this locus. We found that these CTCF sites share a consensus motif distinct from other CTCF sites in the mouse genome. Additionally, we could divide these CTCF sites into three categories: intergenic sites remote from any coding element, upstream sites present within 8 kb of the V_H_-leader exon, and recombination signal sequence (RSS)-associated sites characteristically located at a fixed distance (∼18 bp) downstream of the RSS. We noted that the intergenic and upstream sites are located in the distal portion of the V_H_ locus, whereas the RSS-associated sites are located in the D_H_-proximal region. Computational analysis indicated that the prevalence of CTCF-binding sites at the IgH locus is evolutionarily conserved. In all species analyzed, these sites exhibit a striking strand-orientation bias, with >98% of the murine sites being present in one orientation with respect to V_H_ gene transcription. Electrophoretic mobility shift and enhancer-blocking assays and ChIP–chip analysis confirmed CTCF binding to these sites both *in vitro* and *in vivo*.

## Introduction

During the vertebrate adaptive immune response, B and T cells play an essential role in clearing pathogens from the host organism. Specific recognition of these pathogens relies on the strikingly diverse binding specificities encoded by the antigen receptors, the B-cell receptor (BCR)^10^ and the T-cell receptor (TCR), respectively, expressed on the surface of these lymphoid cells. Antigen receptor genes encoding receptors with distinct specificities are generated from component gene segments, termed variable (V), diversity (D), and joining (J) gene segments, via the V(D)J recombination process. V(D)J recombination is initiated when the V(D)J recombinase, a heterotetrameric complex containing two RAG1 and two RAG2 subunits (1, 2), introduces DNA double-strand breaks at the junctions between the two gene segments and their flanking recombination signal sequences (RSS). The recombination reaction is completed by the ubiquitously expressed nonhomologous end-joining machinery, which joins the two coding ends to form a complete coding sequence, while in parallel joining the two signal ends to each other.

The assembly of BCR and TCR genes via V(D)J recombination is tightly regulated *in vivo*. Rearrangement events are restricted to particular cell lineages and stages, such that immunoglobulin (Ig) loci are only fully rearranged in B cells, whereas TCR genes are completely assembled only in T cells. In mice, rearrangement occurs in a preferred temporal order, with Ig heavy chain loci rearranging prior to light chain loci. Within a heavy chain locus, D_H_–to–J_H_ joining occurs prior to V_H_–to–DJ_H_ rearrangement. Rearrangement is also allele-restricted, whereas D_H_–to–J_H_ rearrangement occurs on both alleles, only one productive V_H_–to–DJ_H_ rearrangement (to produce a functional heavy-chain gene) and one productive V_L_-to-J_L_ rearrangement (to produce a functional light-chain gene) occur per cell.

To appropriately regulate V(D)J recombination, developing lymphocytes must control the accessibility of the antigen receptor loci to the recombinase machinery. Indeed, a variety of alterations at the antigen receptor loci has been found at different developmental stages. For example, germline transcription, genic and intergenic antisense transcripts, specific histone modifications, nucleosome positioning, monoallelic DNA methylation, nuclear repositioning of antigen receptor alleles, reversible DNA contraction, and chromosomal looping of domains within the receptor loci have all been described (3–8).

Several studies have focused on how trans-acting proteins, including transcription factors (such as Pax5 (9–11) and YY1 (12)), chromatin remodeling complexes (such as SWI/SNF (13, 14)), and histone-modifying enzymes (such as G9a (15) and Ezh2 (16)), contribute to the developmental regulation of V(D)J recombination by modifying the chromatin structure of the antigen receptor loci. CTCF, a ubiquitously-expressed nuclear protein that is involved in many cellular processes, is a particularly interesting transcription factor that has been localized to numerous sites across the murine immunoglobulin heavy chain (IgH) locus (9, 17, 18). Some of these CTCF sites have been functionally analyzed via targeted deletion: a portion of the 3′-regulatory region (3′-RR) of the IgH locus that contains several CTCF- and Pax5-binding sites modestly affects V(D)J recombination and contraction of the locus (19); and a regulatory region located between the V_H_ and D_H_ gene clusters that contain two CTCF-binding elements contributes to the developmental regulation of V(D)J recombination (20, 21). Similarly, reducing or eliminating CTCF expression also appears to affect the immunoglobulin loci: a global reduction in CTCF expression results in increased antisense transcription (17) as well as decreased IgH locus contraction (17, 22), whereas targeted deletion of CTCF increases proximal Vκ gene germline transcription and recombination (23).

Although CTCF has been localized to sites across the murine IgH locus (9, 17, 18), it remains unclear whether it is primarily recruited by direct binding to particular DNA sequences in the IgH locus or whether it is primarily recruited indirectly to chromosomal DNA via protein–protein interactions with other DNA-binding proteins such as Pax5, YY1, or cohesin, each of which has been shown to directly interact with CTCF (24–26). To test the hypothesis that CTCF is recruited to the murine IgH locus by direct recognition of DNA-binding sites, we have used a combination of bioinformatics and ChIP to gain further insight into the sequence determinants of CTCF binding. Analysis of the large number of CTCF DNA-binding sites located throughout the V_H_ domain of the murine IgH locus reveals that these CTCF sites fall into three broad categories: those at a fixed distance (17–19 bp) from RSS elements; those positioned upstream of a V_H_-leader sequence; and those located in intergenic regions not associated with gene segments. When we extended our analysis to the human IgH locus, a similar pattern held. Interestingly, the CTCF-binding sites located throughout the V_H_ domains in both human and mouse have a distinct sub-consensus sequence motif that differs from the generic consensus motif found at CTCF sites throughout the rest of these organisms' genomes. Moreover, the consensus sequence present at the IgH-binding sites differs between RSS-associated and RSS-unassociated CTCF sites. Finally, within the V_H_ domains of both the human and mouse IgH loci, the CTCF-binding sites all share the same orientation.

## Results

### Search for CTCF DNA-binding sites at antigen receptor loci

**Figure 1.**
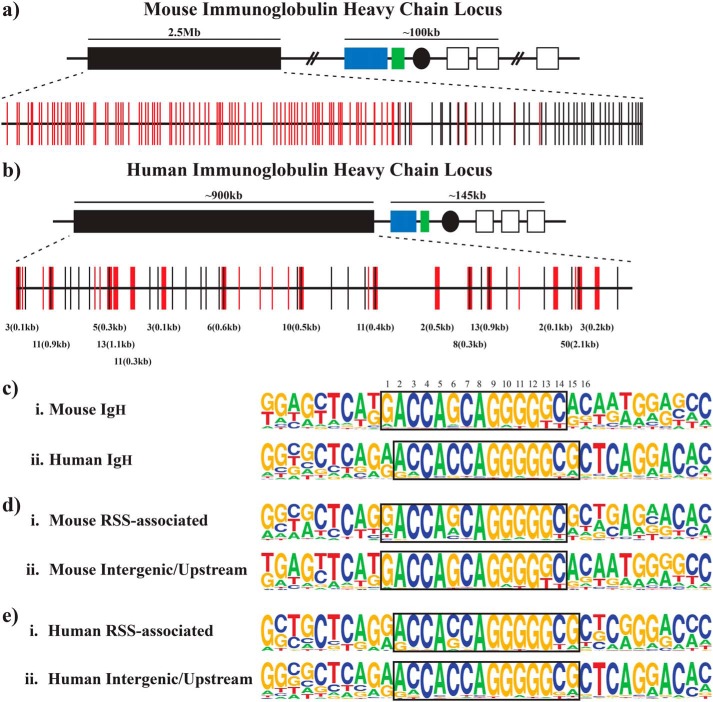
**High density of CTCF sites is found within the V_H_ domains of the murine and human IgH loci.** *a*, schematic of CTCF sites within the murine Ig heavy chain locus. *Red* and *black vertical lines* represent the location of upstream/intergenic and RSS-associated mV_H_ CTCF sites, respectively. The general organizational structure of the murine IgH locus is shown with *rectangles* representing V (*black*), D (*blue*), J (*green*), and constant region (*white*) gene segments. *Black ovals* represent regulatory enhancer elements. *b,* schematic of CTCF sites within the human Ig heavy-chain locus. Diagram is as above. The *numbers below the vertical lines* denote CTCF hot spots with the *first number* indicating the number of putative CTCF sites within each hot spot and the *second number* indicating the length of DNA encompassed within each hot spot. *c*, consensus sequence of the murine and human V_H_ CTCF sites: (*i*) enoLOGOS representation of the frequency of each DNA nucleotide at each position within the murine V_H_ CTCF sites; (*ii*) enoLOGOS representation of the consensus sequence of the human V_H_ CTCF sites. For reference, the consensus CTCF motif at the mouse and human *Igf2/H19* imprinting control regions is CCGCGNGGNGGCAG; the consensus CTCF motif at the chicken β-globin FII 5′HS4 element is CCGCTAGGGGGCAG; and the consensus human CTCF-binding site based on genome-wide ChIP-chip analysis (47) is CCASYAGRKGGCRS. *Boxes* highlight the core CTCF motif as referred to in the text, with nucleotide numbering provided above. *d*, comparison of the consensus sequences of the murine RSS-associated (*i*) and upstream/intergenic (*ii*) CTCF-binding sites. *e*, comparison of the consensus sequences of the human RSS-associated (*i*) and upstream/intergenic (*ii*) CTCF-binding sites.

To identify potential CTCF-binding sites at the IgH locus, we performed a computational search for CTCF sites at the murine IgH locus. Initially, the CTCF-binding site consensus sequence from the chicken β-globin 5′HS4 FII element, 5′-CCGCTAGGGGGCAG-3′ (27), was used to search for CTCF sites at the murine IgH locus. Allowing for two mismatches from the consensus sequence, this search revealed 96 putative CTCF-binding sites within the locus. Using these 96 sites, we identified a new murine IgH CTCF consensus sequence, “mV_H_–CTCF” (5′-GACCAGCAGGGGGC-3′). We then repeated our computational search, allowing for two mismatches from mV_H_–CTCF. This search identified a total of 144 putative CTCF-binding sites in the murine IgH locus (Table S1), of which 138 were located within the V_H_ domain of the locus (Fig. 1*a* and see below). Of the sites not located within the V_H_ domain, one is in an intergenic region within the D_H_ segment cluster, two are in the constant region domain, and three are in the 3′-RR, as reported previously (28). Interestingly, our search did not identify CTCF-binding elements 1 (CBE1) and 2 (CBE2) (20, 21, 29), because the CTCF-binding motif in CBE1 and CBE2 each has six mismatches from mV_H_–CTCF.

Although previous studies have identified CTCF-binding sites at the murine IgH locus (9, 17, 18), it remained unclear whether these CTCF sites are conserved throughout evolution. To address this question, we used the mV_H_–CTCF sequence to perform a similar computational search of the human IgH locus. This search identified 131 putative CTCF-binding sites. A new consensus sequence derived from the human IgH-CTCF sites, “hV_H_–CTCF” (5′-ACCACCAGGGGGCG-3′), contained minor differences from the mouse sequence at the 5′ and 3′ ends of the motif. Repeating our computational search of the human IgH locus with hV_H_–CTCF increased the total number of sites identified in the human IgH locus to 188 (Table S2), of which 183 are within the V_H_ region (Fig. 1*b*). The density of CTCF-binding sites is much higher within the human and murine IgH loci than in the rest of the human and mouse genomes (Table S3). Repeating this search on the partial genomic sequences available for chimpanzee and rabbit also revealed the presence of numerous CTCF sites in the V_H_ region, suggesting that the prevalence of CTCF sites in the V_H_ domain is indeed evolutionarily conserved (Table S3).

Given that CTCF sites have been identified at the murine Igκ (18, 23) and TCRα (30) loci, we next asked whether CTCF-binding sites are equally abundant at the other antigen receptor loci, Igκ, Igλ, TCRβ, TCRαδ, and TCRγ, in humans and mice, and whether they share the V_H_–CTCF motif. Repeating our computational search of the murine and human antigen receptor loci with mV_H_–CTCF and hV_H_–CTCF, respectively, we identified a number of putative CTCF-binding sites in each of the antigen receptor loci, but they were much less abundant than in either the murine or human IgH loci (Table S3).

### Conserved orientation of CTCF-binding sites

Analyzing the murine IgH locus, we noticed that all but two of the 147 identified CTCF sites at the murine V_H_ domain are present in the same preferred orientation, consistent with previous findings (31). This same orientation bias is observed for the human (174/183) and chimpanzee (108/118) V_H_–CTCF sites, suggesting that the orientation of CTCF-binding sites within the V_H_ domain of the IgH locus is evolutionarily conserved and presumably functionally important, as suggested previously (32).

### Distinct locations of CTCF sites

Locations of CTCF sites within the murine IgH locus are far from random. Within the murine V_H_ domain, we found two classes of CTCF-binding sites: RSS-associated sites (30% of all CTCF-binding sites in this region; Fig. 1*a*, *black vertical lines*) and RSS-unassociated sites (70% of all binding sites in this region; Fig. 1*a*, *red vertical lines*). The overwhelming majority of the RSS-associated CTCF-binding sites are positioned with their consensus core-binding sequence (5′-GACCAGCAGGGGGC-3′) located precisely 17–19 bp downstream of the nearby V_H_ RSS nonamer, with only three sites violating this rule (see Table S1). Notably, although the sequence of the RSS-associated CTCF-binding sites is as highly conserved as the RSS itself, it is much more conserved than the sequence of the 17–19 bp between the RSS and CTCF sites. Thus, the length of this RSS–CTCF spacer region appears to be conserved even though the sequence itself is not.

In contrast, the positions of the RSS-unassociated CTCF-binding sites are much more variable with respect to nearby V_H_ gene segments. Upon closer analysis, the RSS-unassociated sites can be further divided into two subclasses: intergenic sites, which are not in close association with a V_H_ gene segment (12% of all CTCF-binding sites), and upstream sites, which are positioned upstream of a V_H_ leader exon (58% of all CTCF-binding sites). The upstream sites can be further subdivided by their distance from the nearest V_H_ leader exon, with subsets located ∼800 bp, 2–3 kb, or 5–6 kb away.

Within the human and murine IgH locus, the V_H_-coding segments can be divided into families based on DNA sequence similarity (greater than 80% identity with all others in the family) and then further subdivided into three clans of closely related families. CTCF sites are found adjacent to RSS elements from 11 of the 16 murine and 3 of the 8 human gene segment families. Four of the five murine V_H_ gene segment families that lack RSS-associated CTCF sites (J558, SM7, Vgam3.8, and VH15) are in the same evolutionarily conserved clan (defined as group 1 (33)), whereas the fifth (3609) is from group 2. Moreover, all of the functional members of the second and fourth largest murine V_H_ families, 7183 and Q52, have an RSS-associated CTCF site, but the nonfunctional ones typically do not. Examining a phylogenetic tree of the V_H_ gene segments (33), and focusing on just those branches that contained RSS-associated CTCF sites, we found only three V_H_ gene segments (VH11.1.48, VH 11.2.53, and VH12.1.78) that lacked an identifiable RSS-associated CTCF site. Closer inspection revealed the presence of a plausible CTCF site 19 bp away from each of these three RSSs, but with greater deviations from the consensus. Two of these sites have three mismatches, whereas one has four, but the sites contain a 4/5 or 5/5 match with the five central guanines of the core CTCF site, and both sites have only a 1- or 2-bp mismatch from the human sequence (Table S1). Thus, including these sites, there are 141 putative CTCF sites in the murine V_H_ region and 147 overall. Strikingly, the presence of an RSS-associated CTCF site can be predicted based on an evolutionary tree constructed with a sequence that does not include the CTCF-containing regions (*i.e.* just the V_H_-coding regions and RSSs), suggesting that these CTCF sites may play an evolutionarily significant role at these murine V_H_ gene segments.

In the human IgH locus, as in the murine locus, CTCF sites are also found either in close association with RSS sequences or at upstream/intergenic positions. For the majority of the human RSS-associated CTCF sites, the core CTCF-binding sequence is generally located a fixed distance away from the RSS, either 19 or 48 bp downstream of the RSS nonamer. Interestingly, the human homologs of the mouse proximal V_H_ gene segments (which have RSS-associated CTCF sites) also have RSS-associated CTCF-binding sites. Additionally, whereas the non-RSS–associated CTCF sites in the murine V_H_ domain are present as individual sites, the non-RSS–associated CTCF sites in the human V_H_ domain are present as “hot spots” of multiple sites that vary in density, from three sites within 100 bp of each other to 50 sites within 2.1 kb of each other (see Fig. 1*b*).

### RSS-associated and intergenic/upstream CTCF sites are restricted to different regions of the murine V_H_ domain

The murine V_H_ domain consists of 195 V_H_ gene segments spanning 2.5 Mb of DNA. Notably, the RSS-associated CTCF sites are sequestered in the D-proximal 900 kb of the V_H_ region, whereas the intergenic CTCF sites are found in the D-distal 1.6 Mb (Fig. 1*a*), with some interspersion of RSS-associated and intergenic/upstream CTCF sites at the approximate interface between the “proximal” and “distal” regions. A number of studies have revealed distinct patterns in the regulation of recombination of these two regions (see under “Discussion”). The approximate border between distal and proximal regions, as defined by these functional studies, is mirrored by the transition point between the domain containing upstream/intergenic sites and the region composed exclusively of RSS-associated CTCF sites (Fig. 1*a*). No such distinct regulatory domains have been identified within the human V_H_ domain, and in the human V_H_ region intergenic/upstream and RSS-associated CTCF sites are intermingled, reflecting substantial intermingling of V_H_ clans.

### Murine intergenic/upstream CTCF sites generally lack a site for CpG methylation

Although mouse and human V_H_ consensus CTCF sites are similar to each other and to the murine *Igf2/H19* CTCF consensus site and the chicken β-globin FII element, one difference is apparent: the CpG dinucleotides crucial for regulation in the Igf2/H19 and the chicken β-globin FII element are absent in the mouse and human V_H_ consensus CTCF motifs (see Fig. 1*c*, nucleotides marked as *4,5* and *6,7* where *numbering* refers to murine consensus shown as a *boxed region*). These CpG dinucleotides are subject to differential methylation that regulates the binding of CTCF to its target site, conferring monoallelic expression at the Igf2/H19 locus (34–37) and developmentally regulated β-globin gene expression (38). CTCF binding at the X-inactivation locus is also regulated by allele-specific CpG methylation (39). Although CpG sites are lacking at positions 4/5 and 6/7, a CpG site is present instead at position 14/15 of the V_H_ CTCF motif in ∼50% of the murine sites and 60% of the human sites (Fig. 1*c*).

Although both intergenic and RSS-associated sites were identified by searches with the same motif (allowing two mismatches), we asked whether conserved differences between these two types of sites would allow for further subdivision. Consensus motifs for the CTCF DNA-binding sites were determined independently for intergenic/upstream and RSS-associated sites for both the mouse and human IgH sites, using the Energy Normalized Logo (enoLOGOS) system (Fig. 1, *d* and *e*). A CpG target is present at position 14/15 for 59% of the human intergenic and RSS-associated consensus motifs (Fig. 1*e*). A CpG site at this position is also present in approximately half of the murine RSS-associated CTCF sites; the other half lacks any CpG dinucleotides (Fig. 1*d*). With only two of the murine upstream/intergenic CTCF sites having a CpG dinucleotide (Fig. 1*d*), the binding to these sites as a class cannot be regulated by differential CpG methylation.

### IgH CTCF sites are bound in vitro by CTCF protein

**Figure 2.**
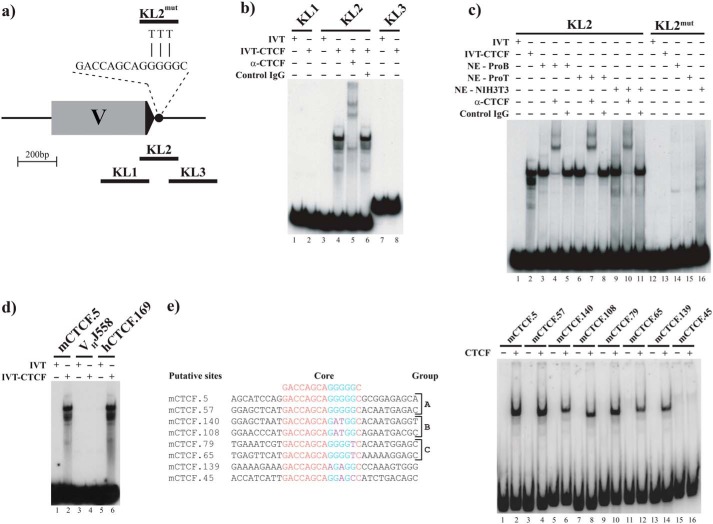
**CTCF binds to the putative V_H_ CTCF sites *in vitro*.** *a*, schematic of the 7183.2.3 genomic segment drawn to scale depicting the location of the DNA probes used in the EMSAs in *b* and *c*. VH7183.2.3 segment–coding sequence (*gray rectangle*), RSS (*black triangle*), and mCTCF.5 site (*black circle*) are shown. The sequence of the targeted DNA transversion of the three central guanine residues within the CTCF present in probe KL2^mut^ is shown. *b*, probe KL2, which encompasses mCTCF.5, is bound by IVT-CTCF and super-shifted by an α-CTCF antibody (CTCF-IgG). *IVT*: *in vitro* translation reaction lacking specific cDNA. *c*, point mutations in mCTCF.5 disrupt binding of IVT-CTCF as well as endogenous CTCF present in nuclear extracts from pro-B (*NE-ProB*), pro T (*NE-ProT*), and NIH3T3 (*NE-NIH3T3*) cells. *d*, human V_H_ CTCF site is bound by CTCF. Murine and human EMSA probes are as indicated. No binding to the region surrounding murine V_H_ segment J558.69.170 (V_H_J558) is observed, indicating the absence of a cryptic CTCF site. *e*, CTCF binds to distinct subclasses of mCTCF sites. *Left panel* shows sequences of representative members of distinct groups of CTCF sites containing the same substitutions within the central G pentad, but differing in sequences flanking the core CTCF site. *Right panel* shows EMSA of the corresponding labeled DNA probes.

Having identified these putative murine IgH CTCF sites *in silico*, we next wanted to ask whether these sites are *bona fide* CTCF-binding sites. To address this question, we first employed electrophoretic mobility shift assays (EMSA) to test whether these DNA sequences can be bound by CTCF *in vitro*, as has been done previously for other CTCF sites (27, 38, 39). Using three partially overlapping 200-bp DNA probes encompassing a portion of the 7183.2.3 gene segment and its RSS-associated CTCF site (mCTCF.5) (Fig. 2*a*), we found that the probe (KL2) containing the RSS-associated CTCF site (mCTCF.5) flanking the 7182.2.3 gene segment was shifted by both *in vitro*-transcribed/translated CTCF (IVT-CTCF) (Fig. 2*b*, *lane 4*) and nuclear extracts from pro-B, pro-T, and NIH3T3 cells (Fig. 2*c*, *lanes 3, 6,* and *9*), whereas the adjacent probes (KL1 and KL3), which lacked the CTCF-binding sit, were not shifted (Fig. 2*b*, *lanes 2* and *8*). The shifted bands we observed using IVT-CTCF and nuclear extracts were all specifically supershifted by an anti-CTCF antibody (Fig. 2, *b*, compare *lanes 5* and *6*; *c*, compare *lanes 4* and *5, 7* and *8*, and *10* and *11*), confirming that the mobility shift is due to CTCF binding. Moreover, when we mutated the mCTCF.5-binding site by converting three central guanine residues to thymidines (KL2^mut^), we observed no shifted bands for either IVT-CTCF or endogenous CTCF from the nuclear extracts (Fig. 2*c*, *lanes 12–16*), confirming that CTCF was indeed binding to the consensus site we had identified. Thus, mCTCF.5 appears to be a *bona fide* CTCF-binding site.

Although all mV_H_–CTCF sites are extremely similar to each other and to the sequence in KL2, there are subsets of sites with distinct mismatches from the consensus (see groups B and C in Fig. 2*e*). To examine whether these other sites could also bind CTCF in the context of their natural flanking DNA, we performed EMSA with a representative set of consensus and nonconsensus sites derived from RSS-associated, intergenic, and upstream sites. Using substrates that positioned the CTCF site in the center of the fragment, with 40 bp of genomic sequence flanking the site on either side, we found that all but one of these sites were capable of binding CTCF, suggesting that the murine V_H_ CTCF sites can generally function as *in vitro* CTCF-binding sites (Fig. 2*e*). Furthermore, when we tested one of the human V_H_ CTCF sites (hCTCF.169), we found that it could also be bound by IVT-CTCF (Fig. 2*d*, *lane 6*). However, no binding was detected to probes derived from the murine VHJ558 gene segments (Fig. 2*d*, *lane 4*), indicating that there are no noncanonical CTCF-binding sites associated with the RSS sequence of the distal V_H_ gene segments.

### mV_H_–CTCF-binding sequences from IgH exhibit enhancer-blocking activity

**Figure 3.**
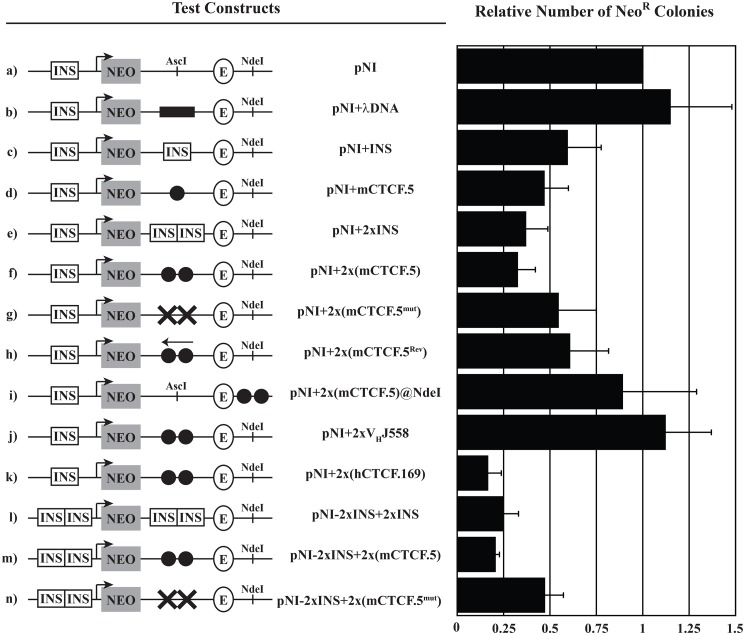
**CTCF sites located within IgH loci possess strong enhancer-blocking activity.** The constructs used in the enhancer-blocking assay are shown on the *left*, and the extent of enhancer blocking (number of neomycin resistant colonies normalized to the backbone vector *pNI*) is shown on the *right*. Data shown represent the average ± S.D. of at least two independent enhancer-blocking experiments. The chicken β-globin 5′HS4 INS, the murine β-globin 5′HS2 locus control element (*E*), the neomycin resistance cassette (*NEO*) driven by the human γ-globin promoter (*arrow*) and restriction enzyme sites used for cloning (AscI and NdeI) are shown. The 2.3-kb λ phage DNA fragment is indicated as a *black rectangle,* and *black circles* refer to the indicated V_H_ gene-segment fragment encompassing the downstream CTCF site. The presence of a mutated CTCF site is indicated by a *black X*, and a V_H_ gene segment that is oriented in the antisense direction is indicated by a *left-facing arrow*.

Previous studies have established that CTCF binding generally confers the enhancer-blocking activity observed in many vertebrate insulator elements (21, 27, 34, 37). Therefore, to further confirm that the murine and human V_H_ CTCF sites we identified are *bona fide* CTCF sites, we utilized a standard enhancer-blocking assay (27, 37). DNA fragments containing mCTCF.5 exhibited slightly stronger enhancer-blocking activity than the classical insulator element (INS) from the chicken β-globin locus (compare Fig. 3, *c versus d*). As with the β-globin INS, inclusion of a second copy of mCTCF.5 increased the enhancer-blocking activity (compare Fig. 3, *e versus f* and *l versus m*). As is often observed with CTCF sites (27), reversing the orientation of mCTCF.5 dramatically reduced the enhancer-blocking activity (compare Fig. 3, *f* and *h*), indicating that this activity is orientation-dependent. As expected, expression of the neomycin reporter was only blocked when mCTCF.5 was positioned between the enhancer and the promoter (Fig. 3*i*), confirming that this element is an enhancer-blocker rather than a DNA silencer. Consistent with the results of our gel-shift analysis, mutating three of the central G residues in the core portion of the CTCF-binding site reduced enhancer-blocking activity (Fig. 3, *g* and *n*). Finally, we also observed potent enhancer-blocking activity by a DNA fragment containing hCTCF.169 (Fig. 3*k*), but no enhancer-blocking activity by a DNA fragment encompassing a J558 V_H_ gene segment (Fig. 3*j*).

Taken together, these results indicate that both mV_H_–CTCF sites and hV_H_–CTCF sites exhibit potent enhancer-blocking activity in the context of their surrounding sequence, strongly suggesting that they function as CTCF-binding sites in the cell.

### CTCF binds to sites within the endogenous IgH locus

Because a subset of mV_H_–CTCF sites can be stably bound by CTCF *in vitro*, and function as CTCF-binding sites in the context of exogenous DNA fragments *in vivo*, we next performed ChIP followed by quantitative PCR (ChIP-quantitative PCR) in both *ex vivo* cell lines and primary cells to ask whether CTCF binds to endogenous mV_H_–CTCF sites within their normal chromatin context *in vivo*.

**Figure 4.**
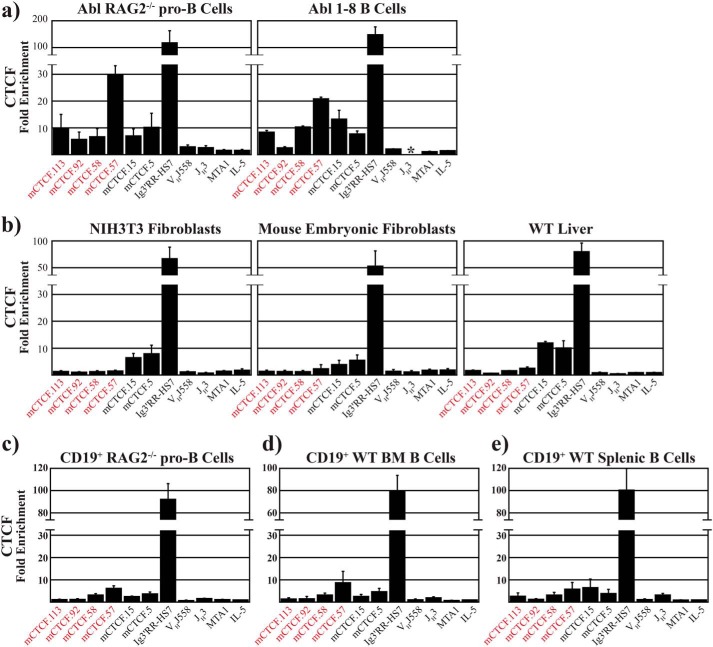
**CTCF binds to its cognate sites *in vivo*.** ChIP with antibodies to CTCF was performed from the indicated cell lines and tissues. Fold-enrichment is shown on the *y* axis. A *break* within the *y* axis of each panel represents a nonlinear jump in fold-enrichment values to accommodate the levels observed from the positive control. All fold-enrichments represent the average ± S.D. of at least three independent chromatin IPs. Primers for the indicated mV_H_ CTCF sites arranged 5′ to 3′ across the IgH locus (with respect to transcription) are described in Table S4 (*red*: upstream/intergenic; *black*: RSS-associated). Primers for the multiple CTCF sites in the 3′-regulatory region of the IgH locus (positive control), the V_H_J558 and J_H_3 gene segments (negative controls), and the MTA1 and IL-5 genes (negative controls) are shown. * indicates that the JH3 DNA in the Abl 1–8 B-cell line has been deleted by V(D)J recombination and, therefore, cannot be assayed.

In both RAG2^−/−^ pro-B cell lines and primary CD19^+^ pro-B cells harvested from the bone marrow of 8-week-old RAG2-deficient mice–where no V(D)J recombination has occurred, and all the antigen receptor loci are in their germline configuration due to the lack of an active recombinase–CTCF was enriched to varying extents at all the intergenic/upstream and RSS-associated sites tested, with maximal V_H_ domain enrichment at mCTCF.57 (Fig. 4*a*, *left panel*, and *c*). Looking at cells from later stages of B-cell development, the 1–8 pre-B–cell line (Fig. 4*a*, *right panel*), CD19^+^ cells from 8-week-old WT bone marrow (Fig. 4*d*), and CD19^+^ WT splenic B cells (Fig. 4*e*), we observed CTCF-binding patterns that were similar to that observed in pro-B cells. However, when we analyzed CTCF binding to the murine IgH locus in nonlymphoid cells, NIH3T3 fibroblasts (Fig. 4*b*, *left panel*), mouse embryonic fibroblasts (Fig. 4*b*, *center panel*), and primary hepatocytes (Fig. 4*b*, *right panel*), we still observed CTCF binding, but the levels of enrichment were lower, and binding was restricted to the RSS-associated D_H_-proximal CTCF sites. Thus, CTCF binding shows distinct patterns in lymphoid *versus* nonlymphoid cells.

### CTCF binds to multiple sites throughout the murine IgH locus

As many of the CTCF sites identified by our computational search are bound by CTCF both *in vitro* and *in vivo*, and because previous studies have observed CTCF-binding sites within the IgH locus (9, 17, 18), we next compared the *in vivo* pattern of CTCF binding across the murine IgH locus in primary CD19^+^ RAG2^−/−^ pro-B cells to our *in silico* predictions. To examine CTCF binding across the entire murine IgH locus, we first isolated CD19^+^ pro-B cells from 8- to 9-week old RAG2^−/−^ mice. Because IL-7 is known to support the growth of primary pro-B cells (24, 31, 40, 41), we expanded these cells in the presence of varying concentrations of recombinant IL-7 before performing ChIP with an α-CTCF antibody (Fig. S1). Next, we took these CD19^+^ RAG2^−/−^ pro-B cells and either expanded the cells for 3 days in the presence of 10 ng/ml of the growth factor IL-7 on OP9 feeder cells and 1 day in the presence of mitomycin C–treated ST2 cells, or we harvested them immediately for ChIP with an α-CTCF antibody. The input DNA and immunoprecipitated DNA were then labeled with Cy3 or Cy5, respectively, hybridized to custom-designed tiling microarrays, and peaks were called by standard bioinformatic analysis (see “Experimental procedures”).

Because the DNA-binding footprint of CTCF is ∼70 bp, adjacent peaks that were very narrowly spaced (<100 bp between them) were combined into a single peak using a PERL script, resulting in a total of 190 CTCF peaks across the murine IgH locus. These peaks ranged in size from 10 to 3498 bp, with an average peak width of 1146 bp.

**Figure 5.**
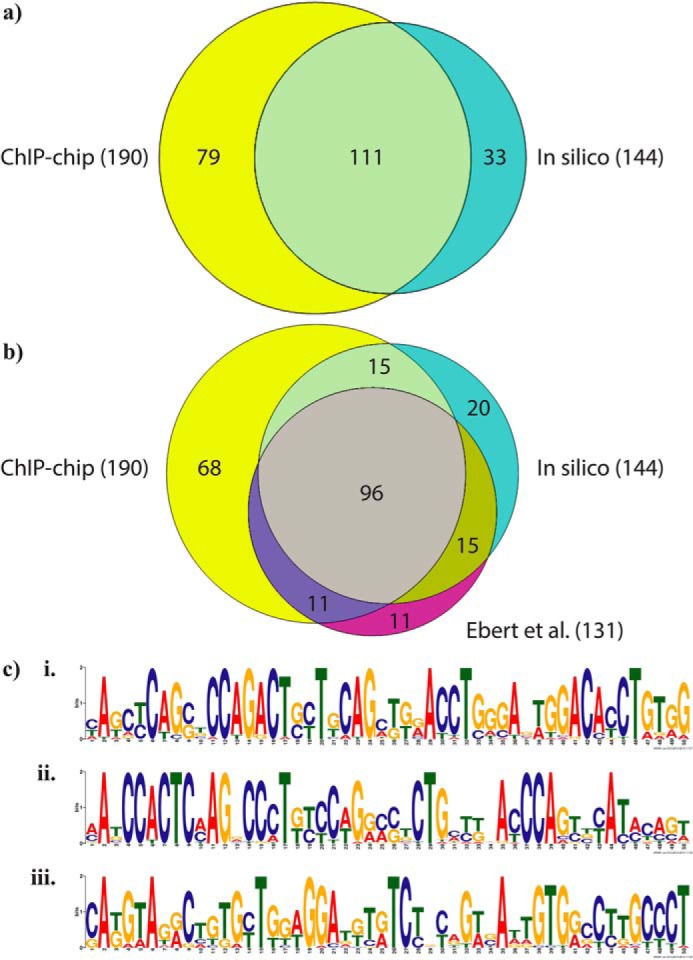
**mV_H_–CTCF consensus motif is a major determinant of CTCF binding at the murine IgH locus.** ChIP with an α-CTCF antibody was performed on CD19^+^ pro-B cells that were isolated from 8- to 9-week-old RAG2^−/−^ mice and expanded for 3 days in the presence of IL-7 (10 ng/ml). Cy3-labeled input DNA and Cy5-labeled immunoprecipitated DNA were hybridized to customized tiling DNA microarrays, and peaks were called by the Ringo method (61). *a*, area-proportional Venn diagram showing the overlap between predicted CTCF-binding sites in the V_H_ domain of the murine IgH locus (*cyan*) and observed CTCF peaks (*yellow*). *b*, area-proportional Venn diagram showing the overlap between predicted CTCF-binding sites (*cyan*); CTCF peaks were observed by ChIP-chip (*yellow*), and CTCF peaks were observed previously by ChIP-seq (9). *c*, enoLOGOS representation of three distinct sequence motifs identified by MEME (42) analysis of the 79 CTCF peaks observed by ChIP-chip that did not contain an mV_H_–CTCF consensus motif.

After determining the localization pattern of CTCF across the murine IgH locus in Rag2^−/−^ pro-B cells, we compared the CTCF-binding sites predicted *in silico* to the CTCF-binding sites observed *in vivo*. Of the 144 *in silico*-predicted CTCF-binding sites, 111 were occupied *in vivo* (77%), suggesting that the presence of a CTCF consensus sequence is a major determinant of CTCF binding *in vivo*. Conversely, 58% of the observed CTCF peaks contained an mV_H_–CTCF consensus sequence. Analyzing the overlap between our predicted CTCF-binding sites, the CTCF-binding sites we observed by ChIP-chip, and the CTCF-binding sites previously identified by ChIP-seq in cultured Rag2^−/−^ pro-B cells (9), we found that of the 190 CTCF peaks we identified by ChIP-chip, 107 (56%) overlapped with the CTCF ChIP-seq peaks (Fig. 5*a*; Table S5). Of the 144 putative CTCF-binding sites that matched our predicted consensus motif, 111 (75%) overlapped with the CTCF peaks previously identified by ChIP-seq (Fig. 5*a*).

To learn more about CTCF binding at the peaks that did not overlap with our predicted CTCF consensus motif, we analyzed these 79 sites using MEME (42) to probe for alternative sequence motifs. Three sequence motifs were identified (Fig. 5*c*), none of which bore obvious similarity to the CTCF consensus motif. To determine whether any of these motifs were similar to other known protein-binding site motifs (*e.g.* YY1, cohesin, or nucleophosmin), we compared all three motifs to the JASPAR Vertebrates and UniPROBE Mouse database using Tomtom (42). However, running these three sequence motifs through Tomtom failed to retrieve any statistically significant hits to known motifs in the JASPAR Vertebrates and UniPROBE Mouse database.

**Figure 6.**
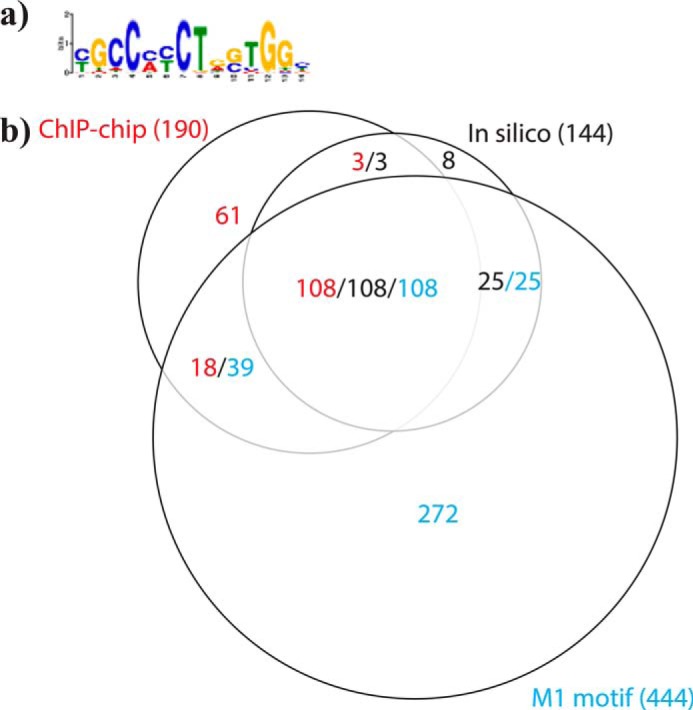
**M1 motif is present in a majority of the predicted and observed CTCF-binding sites at the murine IgH locus.** *a,* enoLOGOS representation of the M1 motif (44). *b*, area-proportional Venn diagram showing the overlap between predicted CTCF sites (*black text*), observed CTCF peaks (*red text*), and M1 motif (*cyan*).

Finally, because previous studies have identified a two-part CTCF-binding motif consisting of a fairly well-conserved M1 motif of 20 bp (Fig. 6*a*) adjacent to a less well-conserved M2 motif of 9 bp (43, 44), we analyzed the overlap between our predicted murine V_H_ CTCF-binding sites, the CTCF-binding sites we observed by ChIP-chip, and predicted M1 sites. Of the 190 CTCF peaks we identified by ChIP-chip, 126 (66%) contained a predicted M1 motif (Fig. 6*b*). Of the 144 putative CTCF-binding sites that matched our predicted consensus motif, 133 (92%) contained a predicted M1 motif (Fig. 6*b*). Of the 79 CTCF ChIP-chip peaks that did not overlap with our predicted CTCF consensus motif, 18 (23%) contained a predicted M1 motif (Fig. 6*b*).

## Discussion

### Molecular determinants of CTCF binding at IgH locus

Although previous studies have analyzed CTCF occupancy at the IgH locus (9, 17, 18), the mechanism by which CTCF is recruited to the IgH locus during B-cell development remains unclear. Does CTCF bind directly to particular DNA sequences in the IgH locus when these sequences become accessible, or is it being recruited indirectly via protein–protein interactions with other DNA-binding proteins, such as YY1 (12, 25), cohesin (18, 45), or Pax5 (9, 10)? Here, we find that the majority of CTCF-occupied sites overlap with a computationally-identifiable sub-consensus motif, mV_H_–CTCF (5′-GACCAGCAGGGGGC-3′), that is distinct from the generic CTCF consensus motif that is found elsewhere in the mouse genome. This CTCF sub-consensus motif is unique to the V domain of the IgH locus, highly conserved between binding sites within the locus, and displays far more sequence conservation than the surrounding sequences. Thus, there was likely a strong evolutionary pressure to maintain this specific version of the CTCF-binding site, despite the known ability of CTCF to bind to degenerate sequences. Moreover, we find that CTCF can directly bind to these sites *in vitro*, suggesting that although other proteins may help to stabilize CTCF once it is bound, CTCF is likely recruited to the IgH locus directly via its sequence-specific DNA-binding activity. Given the differences between mV_H_–CTCF and previously identified CTCF-binding site consensus motifs (27, 34, 39), and given that distinct functions have been ascribed to individual zinc fingers within CTCF (38, 46), it is tempting to speculate that CTCF uses a distinct combination of its 11 zinc fingers to bind mV_H_–CTCF, as compared with other CTCF sites located throughout the mouse genome, thereby leaving a similarly distinct combination of its zinc fingers available for protein–protein interactions with other known CTCF-interacting proteins such as cohesin (18, 45), YY1 (12, 25), or the lymphoid-specific protein Pax5 (9, 10). Further studies will be required to test this hypothesis.

### Evolutionary conservation of numerous CTCF-binding sites across the IgH locus

The 2.5-Mb murine IgH locus contains an extraordinarily high number of CTCF sites (this work and see Refs. 9, 17, 18, 20), especially as compared with the number of CTCF sites found at the other murine antigen receptor loci and several orders of magnitude greater than the mouse genome generally (44). Similarly, the 1.25-Mb human IgH locus contains a remarkably large number of CTCF-binding sites, with a density of sites that is an order of magnitude greater than the other human antigen receptor loci (this study), and several orders of magnitude greater than the human genome generally (47). Other studies using computational methods or genome-wide ChIP analysis have also identified CTCF sites at the human TCRβ, TCRα/δ, IgH, Igκ, and Igλ loci (9, 18, 30, 47, 48). Although the precise numbers of sites vary somewhat between these studies, possibly reflecting either the different search sequences, the specific cell type being examined in the ChIP studies, the probe content of the microarrays, or the peak-calling algorithms, the high density of CTCF-binding sites at the IgH locus is striking, particularly because it is evolutionarily conserved in mice, rabbits, chimpanzees, and humans (this study). The high density of conserved CTCF-binding sites underscores the likely importance of CTCF in regulation of antigen receptor loci, consistent with recent studies (17, 20, 22, 24, 49). However, the exact function(s) of these multiple sites at the IgH locus remains unclear (see below).

### Distinct classes of CTCF-binding sites within murine IgH locus

Using our computational consensus motif-based approach, we not only identified a similar number of CTCF-binding sites within the murine IgH locus, but we also discovered two distinct classes of CTCF-binding sites: RSS-associated sites that are located ∼19 bp downstream of the nearest RSS, and RSS-unassociated sites that are located at least 800 bp away from the nearest RSS (17). We note that the RSS-associated CTCF sites are all located within the D_H_-proximal region of the V_H_ domain, whereas the RSS-unassociated CTCF sites are located in the D_H_-distal region of the V_H_ domain. In addition, it is intriguing that for the RSS-associated sites, the distance from the CTCF site to RSS is conserved (∼2 turns of the double helix), even though the intervening DNA sequence is not, suggesting that the RSS–CTCF distance is functionally significant. Although a previous study noted that CTCF sites in the proximal half of the V_H_ locus were within 150 bp of the RSSs (17), we find a much tighter association between the RSS-associated CTCF sites and the adjacent RSSs. It is noteworthy that RSS-associated CTCF sites are also positioned a fixed distance from their associated RSSs in humans (∼2 or 4 turns of the double helix) and other species. Because the accessibility of the D_H_-distal and D_H_-proximal regions of the V_H_ domain is known to be differentially regulated during B-cell development, we suggest that the RSS-associated CTCF sites in the D_H_-proximal region likely have a function that is distinct from the RSS-unassociated CTCF sites in the D_H_-distal region of the V_H_ domain. Indeed, we have recently shown that CTCF binding to these RSS-associated sites is highly predictive of high-frequency recombination among D_H_-proximal V gene segments (49). Furthermore, given that the distance from the RSS-associated CTCF sites to their associated RSSs is either two or four turns of the double helix in mice and humans, it is tempting to speculate that CTCF may be directly influencing the activity of the RAG1/2 proteins at these gene segments. Future studies will test this hypothesis.

Additionally, whereas CTCF sites at the Igf2/H19 locus (34–37), β-globin locus (38), the X-inactivation locus (39), and the IgH super-anchor (31) are all regulated by CpG methylation, only 50% of the murine RSS-associated CTCF sites contain CpG motifs, suggesting that binding to a large fraction of these sites is either not regulated or is regulated in a CpG-independent manner. Moreover, only two of the murine upstream/intergenic CTCF sites contain a CpG dinucleotide, indicating that CTCF binding to these sites cannot be regulated by CpG methylation. Thus, the differential binding observed in distinct cell types may reflect distinct chromatin structure that occurs independently of (and therefore prior to) CTCF binding. Furthermore, in much the same way that the D_H_-proximal region CTCF sites (which are RSS-associated) may have a distinct function from the D_H_-distal region CTCF sites (which are RSS-unassociated), CTCF binding to these two classes of sites may also be regulated in different ways. Further experiments will be required to explore the differential function and regulation of these CTCF sites within the V_H_ domain of the murine immunoglobulin heavy-chain locus.

### Conserved orientation of CTCF sites with V_H_ domain of IgH locus

Given the large number of CTCF sites within the murine IgH locus, it is striking that over 98% of these sites are present in the same orientation. Because CTCF has been found to affect chromosomal looping (50, 51), and previous studies have identified CTCF sites with the opposite orientation within the IgH intergenic control region 1 (IGCR1) (29) and the IgH super-anchor (31), it seems likely that one function of the CTCF sites within the V_H_ domain of the IgH locus is to form loops that promote synapsis of DJ_H_ and V_H_ gene segments, as suggested previously (52–54). Moreover, the large number of CTCF sites within the V_H_ domain may allow for competition between sites that synapse to convergent CTCF sites within IGCR1 or the IgH super-anchor, thereby forming distinct chromosomal loop domains that could facilitate linear tracking of RAG1/2, as suggested previously (32). It is worth nothing that CTCF-dependent chromosomal looping has also been implicated in regulating V(D)J recombination at other antigen receptor loci (30, 55). However, because there are several distinct classes of CTCF sites within the murine IgH locus, it seems likely that some of the V_H_ domain CTCF sites are functioning in a looping-independent manner. The RSS-associated CTCF sites in the D_H_-proximal portion of the domain appear to affect the accessibility and activity of the V(D)J recombinase at these gene segments (49). Some of the intergenic non-RSS–associated CTCF sites in the D_H_-distal portion of the domain have been shown to affect the 3D conformation of the locus (20). However, the role of the non-RSS–associated CTCF sites at the interface of the proximal and distal regions is unknown, and it is intriguing to speculate that they function as enhancer blockers that separate the regulation of the proximal and distal regions of the locus. Finally, our studies have revealed that a large class of CTCF-binding sites, namely the upstream sites, conform neither to a conformational nor a local recombinase-activating role. Their conserved spatial distances upstream of V gene segments suggest a possible role in insulating V gene segments from neighboring V gene segments. In any case, understanding the sequence determinants of CTCF binding to the murine IgH locus should facilitate future studies evaluating how IgH locus accessibility regulates CTCF binding as well as the functions that CTCF plays in regulating the recombinational accessibility of V_H_ gene segments during B-cell development.

## Experimental procedures

### Mice and cell culture

Animal experiments and procedures were approved by the Institutional Animal Care and Use Committee of Massachusetts General Hospital. WT and RAG2^−/−^ mice were obtained from Taconic Farms and were bred and maintained in HPP-free animal facilities at Massachusetts General Hospital. Pro-B cells were recovered from femoral bone marrow suspensions derived from 8-week-old mice by positive enrichment of CD19^+^ cells using MACS magnetic separation (Miltenyi Biotec). A portion of these cells was placed into culture in the presence of IL-7 prior to harvesting for chromatin IP, whereas chromatin was prepared from the remaining cells and frozen to permit immunoprecipitation in parallel with material recovered from cultured cells. See the supporting information for additional information about culturing conditions for CD19^+^ cells.

WT livers and spleens were forced through a 19-gauge needle and passed through a sterile mesh filter to generate single-cell suspensions. The cells were washed, and splenic B cells were collected by positive enrichment of CD19^+^ cells using MACS magnetic separation.

### Cell lines

RAG2^−/−^ Abelson-transformed pro-B cells, RAG1^−/−^ p53^−/−^ pro T cells, and Abelson-transformed 1–8 B cells were maintained in RPMI 1640 medium supplemented with 20% fetal bovine serum and 0.05 mm 2-mercaptoethanol. NIH3T3 fibroblast cells and mouse embryonic fibroblasts were maintained in Dulbecco's modified Eagle's medium supplemented with 10% calf serum. Human erythroleukemia K562 cells (a gift from Jeannie Lee) were cultured in Iscove's modified Dulbecco's medium supplemented with 10% fetal bovine serum.

### Sequence alignments

Annotated genomic sequence spanning antigen receptor loci was obtained from GenBank^TM^ (see supporting information for accession numbers). Vseg elements for the IgH loci of chimpanzee (NW_001224639.1), chicken (NW_001477447.1 and NW_001484419.1), and dog (NW_876328.1) were identified by tblastx using mouse sequences as the blast query. Searches for CTCF DNA-binding sites and sequence alignments were performed using MacVector version 7.2 and EMBOSS version 4.1.0.

### Electrophoretic mobility shift assays

DNA probes were obtained by PCR amplification from either human HeLa cell genomic DNA or murine pro-B–cell genomic DNA (see Table S4 for primer sequences), gel-purified, and sequenced. All probes were 5′-end–labeled with [^32^P]ATP as described (56). The 250-bp probes KL1 and KL3 each overlap with the 200-bp KL2 probe by 50 bp. IVT-CTCF was prepared from *pCTCF* (a gift from Jeannie Lee) using the TnT Coupled Reticulocyte Lysate System (Promega). Nuclear extracts were prepared from ∼1 × 10^8^ pro-B–cells, pro-T–cells, or NIH3T3 cells as described (57). CTCF protein was purified from a HeLa cell line that stably expresses a double-tagged FLAG–HA–hCTCF transgene as described (58). See supporting information for additional details.

### Enhancer blocking assay

K562 cell transfections and colony assays were performed as described previously (59). See supporting information for a detailed description of the methodology.

### Chromatin immunoprecipitation

Chromatin immunoprecipitations were performed as described (60) with 30 μl of anti-CTCF antibody (Upstate Biotechnology) and analyzed by real-time PCR with SYBR Green or TaqMan probes or by hybridization to custom DNA microarrays. For additional information about ChIP methodology, supporting information. For primer and probe sequences see Table S4.

### Microarray hybridization and processing

Tiling genomic DNA microarrays were custom-designed (NimbleGen Systems, Inc.) based on the mm9 release of the IgH locus sequence (murine chr12: 114,341,024–117,349,200). The 50-mer probes were selected every 20 bases with no repeat masking on both the top and bottom strands. Three replicates for each strand were spotted on the array. Genomic DNA and CTCF ChIP DNA were labeled with Cy3 and Cy5, respectively, and hybridized to the array by the manufacturer.

### Computational analysis

The Ringo method (61) was implemented as a Bioconductor package to identify CTCF peaks from the ChIP-microarray data. A position weight matrix for the M1 motif (44) was downloaded from CTCFBSDB database (62, 63). The search for the M1 motif matches across the regions of interest was performed using FIMO (64) with default parameters.

## Author contributions

D. N. C., Y. N., K. B. M., A. L. W., and M. A. O. conceptualization; D. N. C., Y. N., C. C., K. B. M., A. L. W., C. M. J., J. W. M., Y. W., R. S., A. E. C., A. G. W. M., and M. A. O. formal analysis; D. N. C., R. S., A. E. C., A. G. W. M., and M. A. O. supervision; D. N. C. and M. A. O. funding acquisition; D. N. C., Y. N., C. C., K. B. M., and A. L. W. validation; D. N. C., Y. N., C. C., K. B. M., A. L. W., C. M. J., J. W. M., A. G. W. M., and M. A. O. investigation; D. N. C., Y. N., C. C., K. B. M., A. L. W., C. M. J., J. W. M., Y. W., R. S., A. E. C., A. G. W. M., and M. A. O. methodology; D. N. C., Y. N., K. B. M., A. L. W., A. G. W. M., and M. A. O. writing-original draft; D. N. C. and M. A. O. project administration; D. N. C., Y. N., C. C., K. B. M., A. L. W., C. M. J., J. W. M., Y. W., R. S., A. E. C., A. G. W. M., and M. A. O. writing-review and editing; A. L. W., C. M. J., J. W. M., Y. W., R. S., A. E. C., and A. G. W. M. resources; J. W. M., Y. W., R. S., A. E. C., A. G. W. M., and M. A. O. data curation; J. W. M., Y. W., R. S., A. E. C., and A. G. W. M. software.

## Supplementary Material

Supporting Information

## Footnotes

10 The abbreviations used are:

BCR: B-cell receptor
TCR: T-cell receptor
CTCF: CCCTC-binding factor
IgH: immunoglobulin heavy chain
RSS: recombination signal sequence
IP: immunoprecipitation
IVT-CTCF: *in vitro* transcribed/translated CTCF
m: murine
h: human
3′-RR: 3′-regulatory region
INS: insulator element.
