# Supplementary material for: The murine IgH locus contains a distinct DNA sequence motif for the chromatin regulatory factor CTCF
Source: J Biol Chem. 2019 Jul 8;294(37):13580–92. doi: 10.1074/jbc.RA118.007348 (PMC6746451; doi:10.1074/jbc.RA118.007348)
Supplement: Supporting Information [file supp_294_37_13580__index.html]

The murine IgH locus contains a distinct DNA sequence motif for the chromatin regulatory factor CTCF — CTCF binds evolutionarily-conserved sites in the IgH locus — Supporting Information 

# The murine IgH locus contains a distinct DNA sequence motif for the chromatin regulatory factor CTCF

## Supporting Information

- Supporting Information (to be published online) - Supporting Figures and Tables
- Supplementary methods
