# Supplementary material for: The murine IgH locus contains a distinct DNA sequence motif for the chromatin regulatory factor CTCF
Source: J Biol Chem. 2019 Jul 8;294(37):13580–92. doi: 10.1074/jbc.RA118.007348 (PMC6746451; doi:10.1074/jbc.RA118.007348)
Supplement: Supporting Information [file supp_RA118.007348_142843_2_supp_356964_p70p4w.pdf]

A distinct motif for numerous CTCF binding sites in the murine IgH locus

David N. Ciccone, Yuka Namiki, Changfeng Chen, Katrina B. Morshead, Andrew L. Wood, Colette M. Johnston, John W. Morris, Yanqun Wang, Ruslan Sadreyev, Anne E. Corcoran, Adam G.W. Matthews, and Marjorie A. Oettinger

<u>List of Supporting Information:</u>

1. Figure S1: Pro-B cells cultured in the presence of IL-7 exhibit higher levels of CTCF enrichment throughout the VH domain.
2. Table S1: Murine IgH locus contains 144 putative CTCF binding sites.
3. Table S2: Human IgH locus contains 188 putative CTCF binding sites.
4. Table S3: The prevalence of VH CTCF sites in the IgH domain is evolutionarily conserved.
5. Table S4: PCR primers and Taqman probes used in this study.
6. Table S5: CTCF sites identified by ChIP-chip largely overlap with those predicted computationally.

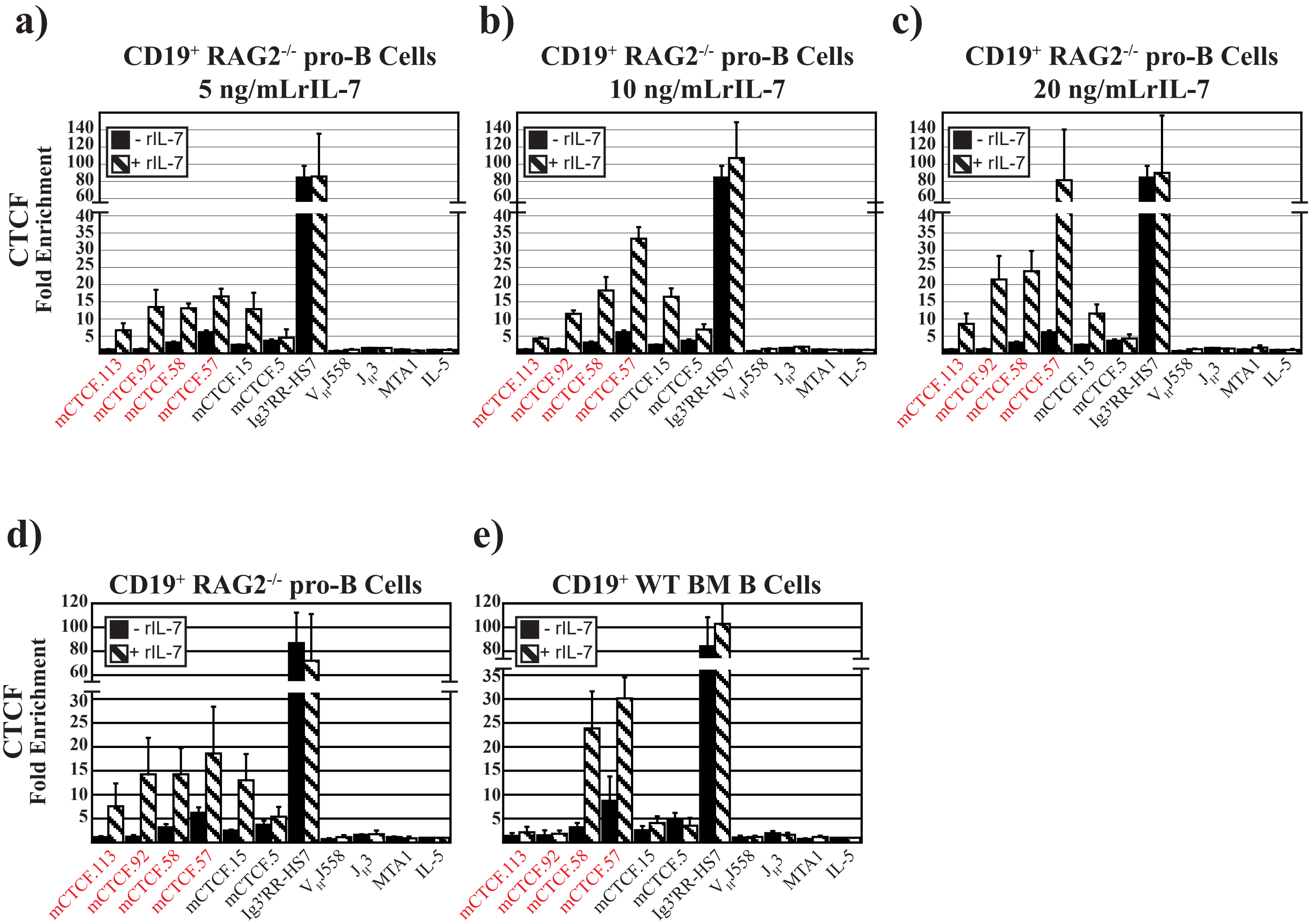
a)
CD19+ RAG2-/- pro-B Cells
5 ng/mLrIL-7
b)
CD19+ RAG2-/- pro-B Cells
10 ng/mLrIL-7
c)
CD19+ RAG2-/- pro-B Cells
20 ng/mLrIL-7
d)
CD19+ RAG2-/- pro-B Cells
e)
CD19+ WT BM B Cells
CTCF
Fold Enrichment
- rIL-7
+ rIL-7
mCTCF.113
mCTCF.92
mCTCF.58
mCTCF.57
mCTCF.15
mCTCF.5
Ig3'RR-HS7
VHJ558
JH3
MTA1
IL-5

Figure S1. Pro-B cells cultured in the presence of IL-7 exhibit higher levels of CTCF enrichment throughout the VH domain. Chromatin immunoprecipitation with antibodies to CTCF was performed from the indicated cells. Fold-enrichment is shown on the y-axis. A break within the y-axis of each panel represents a non-linear jump in fold-enrichment values in order to accommodate the levels observed from the positive control. All fold-enrichments represent the average ± S.D. of at least 3 independent chromatin IPs. Primers for the indicated mV_HCTCF sites arranged 5' to 3' across the IgH locus (with respect to transcription) are described in Table S4. Primers for the multiple CTCF sites in the 3' regulatory region of the IgH locus (positive control), the V_HJ558 and J_H3 gene segments (negative controls), and the MTA1 and IL-5 genes (negative controls) are shown.

Mouse Immunoglobulin Heavy Chain Locus

V_H-CTCF site		Position	Sequence	V_H seg.	Distance
mCTCF	1	114525788-114525801	GACCAGCAATGGGC		
mCTCF	2	114544788-114544801	GACCAGCAATGGGC		
mCTCF	3	114730021-114730034	GACCAGCAAGGGAC		
mCTCF	4	114811068-114811081	GACCAGAAGGAGGC	7183.1pg.1	17
mCTCF	5	114816645-114816658	GACCAGCAGGGGGC	7183.2.3	17
mCTCF	6	114826406-114826419	GACCAGCAGGGGGC	Q52.2.4	20
mCTCF	7	114835587-114835600	GACCAGCAGGGGGC	7183.4.6	17
mCTCF	8	114849323-114849336	AACCAGCAGGGGGC	Q52.3.8	19
mCTCF	9	114863647-114863660	GACCAGCAGGGGGC	7183.7.10	17
mCTCF	10	114891430-114891443	GACCTGCAGGGGGC	Q52.5.13	19
mCTCF	11	114899910-114899923	GACCAGCAGGGGGC	7183.9.15	17
mCTCF	12	114923621-114923634	GACCAGCAGGGGGC	Q52.7.18	19
mCTCF	13	114940265-114940278	GACCAGCAGGGGGC	7183.12.20	17
mCTCF	14	114954809-114954822	AACCAGCAGGGGGC	Q52.8.22	19
mCTCF	15	114974255-114974268	GACCAGCAGGGGGC	7183.14.25	14
mCTCF	16	115000390-115000403	GATGAGCAGGGGGC	7183.16.27	17
mCTCF	17	115007989-115008002	AACCAGCAGGGGGC	Q52.9.29	19
mCTCF	18	115034277-115034290	AACCAGCAGGGGGC	Q52.10.33	19
mCTCF	19	115045453-115045466	GACCAGCAGGGTGT	Q52.11.34	19
mCTCF	20	115064787-115064800	GGCCAGCAGGGGGC	7183.18.35	17
mCTCF	21	115076667-115076680	AACCAGCAGAGGGC	7183.19.36	17
mCTCF	22	115097290-115097303	GACCAGCAGGGGGC	7183.20.37	17
mCTCF	23	115117235-115117248	GACCAGCAGGGGGC	Q52.13.40	19
mCTCF	24	115134547-115134560	GACCAACAGAGGGC	S107.1.42	19
mCTCF	25	115150172-115150185	GACCAACAGGGGGC	S107.2pg.43	19
mCTCF	26	115186421-115186434	AACCACCAGGGGGC	X24.1pg.45	19
mCTCF	27	115202527-115202540	CACCAACAGGGGGC	36-60.1.46	19
mCTCF	28	115209920-115209933	GACCAGGAGGGGGG		
mCTCF	29	115220018-115220031	AACCACCAGGGGGA	VH11.1.48	19
mCTCF	30	115251283-115251296	AACCACCAGGGGGC	X24.2.50	19
mCTCF	31	115271942-115271955	CACCAACAGGGGGC	36-60.2pg.51	19
mCTCF	32	115286380-115286393	AACCACTAGGGGGT	VH11.2.53	19
mCTCF	33	115306952-115306965	GACCAGCAGGGGAC	VH16.1.55	34
mCTCF	34	115308902-115308915	GATCAGGAGGGGGC		
mCTCF	35	115365674-115365687	GACCAGTAGGGGAC	PG.12.60	19
mCTCF	36	115391319-115391332	GACCAACAGGGGGT	S107.3.62	19
mCTCF	37	115434578-115434591	TACCACCAGGGGGC	36-60.3.64	19
mCTCF	38	115460927-115460940	GACCAACAGGGGGC	S107.4.65	19
mCTCF	39	115495974-115495987	GGTCAGCAGGGGGC		
mCTCF	40	115500791-115500804	TACCAACAGGGGGC	36-60.5.67	19
mCTCF	41	115526292-115526305	TACCAACAGGGGGC	36-60.6.70	18
mCTCF	42	115529489-115529502	GGTCAGCAGGGGGC		
mCTCF	43	115558169-115558182	GGCCGGCAGGGGGC	PG.14.73	18
mCTCF	44	115560513-115560526	CACCAACAGGGGGC	36-60.8.74	19
mCTCF	45	115590791-115590804	GACCAGCAGGAGCC		
mCTCF	46	115595900-115595913	GACCATCAGGGGGC	3609N.2.77	19
mCTCF	47	115604659-115604672	AACCAGCAGGTGGT	VH12.1.78	19
mCTCF	48	115629851-115629864	TACCAGCAGGGGGA	J606.1.79	19
mCTCF	49	115711051-115711064	AACCAGCAGGGGGT		
mCTCF	50	115717145-115717158	GACCAGCAGGGGGT	VH10.1.86	18
mCTCF	51	115734143-115734156	GACCAGCAGGAGGC	VH10.2pg.89	18
mCTCF	52	115755306-115755319	AACCAGCAGGGGGT		
mCTCF	53	115761581-115761594	TACCAGCAGGGGGT	VH10.3.91	18

mCTCF 54	115780514-115780527	AACCAGAAGGGGGC
mCTCF 55	115785399-115785412	GACTAGCAGGAGGC
mCTCF 56	115804496-115804509	GACTATCAGGGGGC
mCTCF 57	115825645-115825658	GACCAGCAGGGGGC
mCTCF 58	115853579-115853592	GTCCAGCAGGGGGC
mCTCF 59	115855791-115855804	GACCAGCAGGGGGA
mCTCF 60	115888838-115888851	GACCAGCAGGGGGC
mCTCF 61	115906780-115906793	GACCAGCAGGGGTC
mCTCF 62	115917694-115917707	CACCAGCAGGGGTC
mCTCF 63	115923533-115923546	GACCAGCAGGTGTC
mCTCF 64	115949515-115949528	GACCAGCAGGGGTC
mCTCF 65	115981297-115981310	GACCAGCAGGGGTC
mCTCF 66	115987160-115987173	GACCAGCAGGGGTC
mCTCF 67	115993995-115994008	GACCAGCAGGGGTC
mCTCF 68	116013835-116013848	GACCAGCAGGGGTC
mCTCF 69	116022341-116022354	GACCAGCAGGGGTC
mCTCF 70	116029263-116029276	GACCAGCAGGGGTC
mCTCF 71	116057940-116057953	GACCATCAGGGGTC
mCTCF 72	116070129-116070142	GACCAGCAGGGGTC
mCTCF 73	116075878-116075891	GAACAGCAGGGGTC
mCTCF 74	116084547-116084560	GACCAGCAGGGTTC
mCTCF 75	116094859-116094872	GACCAGCAGGGGTC
mCTCF 76	116120761-116120774	GACCAGCAGGGGTC
mCTCF 77	116137100-116137113	GACCAGCAGGGGTC
mCTCF 78	116155276-116155289	GAACAGCAGGGGTC
mCTCF 79	116166076-116166089	GACCAGCAGGGGTC
mCTCF 80	116177980-116177993	GACCACCAGGGGTC
mCTCF 81	116186799-116186812	GAACAGCAGGGGTC
mCTCF 82	116203351-116203364	GACCAGCAGTGGGC
mCTCF 83	116219897-116219910	GACCAGCAGGGGTC
mCTCF 84	116235227-116235240	GACCAGCAGGGGGC
mCTCF 85	116246846-116246859	GACCAGCAGGGGTC
mCTCF 86	116296343-116296356	GACCAGCACGGGGC
mCTCF 87	116309972-116309985	GACCAGCAGGGGGT
mCTCF 88	116329871-116329884	GACCAGCAGGGGGC
mCTCF 89	116359685-116359698	GACCAGCAGGGGGC
mCTCF 90	116384887-116384900	GACCAGCAGGGGGC
mCTCF 91	116397855-116397868	GACCAGCAGGGGGC
mCTCF 92	116410153-116410166	GACCAGCAGGGGGT
mCTCF 93	116419258-116419271	GACCAGCTGGGGTC
mCTCF 94	116447536-116447549	GACCAGCAGGGGGC
mCTCF 95	116455737-116455750	GACCAGCAGGGGGT
mCTCF 96	116461081-116461094	GACCAGCAGGGGTT
mCTCF 97	116468365-116468378	GACCAGAAGGGGGC
mCTCF 98	116500831-116500844	GACCAGCAGGGGGT
mCTCF 99	116515149-116515162	GACCAGCAGGGGGC
mCTCF 100	116536446-116536459	GACCAGCAGGGGGT
mCTCF 101	116559048-116559061	GACCAGCAGGGGGC
mCTCF 102	116574997-116575010	GACCAGCAGGGGGC
mCTCF 103	116598540-116598553	GACCAGCAGGGGGC
mCTCF 104	116611459-116611472	GACCAGCAGGGGGC
mCTCF 105	116628680-116628693	GACCAGCAGATGGC
mCTCF 106	116645829-116645842	GACCAGCAGGGGTG
mCTCF 107	116652383-116652396	GACCAGCAGGGGGC
mCTCF 108	116691032-116691045	GACCAGCAGATGGC
mCTCF 109	116700448-116700461	GACCAGCAGGGGGC
mCTCF 110	116712707-116712720	GACCCGCAGGGGGT

mCTCF 111	116719784-116719797	GACCAGCTGGGGGC
mCTCF 112	116737295-116737308	GACCAGCAGATGGC
mCTCF 113	116746994-116747007	GACCAGCAGGGGGC
mCTCF 114	116762502-116762515	GACCAGCAGGGGGT
mCTCF 115	116771525-116771538	GACCAGCAGGGGGC
mCTCF 116	116812728-116812741	GACCAGCAGGGGGT
mCTCF 117	116835218-116835231	GACCAGCAGATGGC
mCTCF 118	116843377-116843390	AACCAGCAGGGGGC
mCTCF 119	116853436-116853449	GACAAGCAGGGGGC
mCTCF 120	116862618-116862631	GACCAGCAGGGGGC
mCTCF 121	116894189-116894202	GACCAGCAGGGGGT
mCTCF 122	116902472-116902485	GACCAGGAGGTGGC
mCTCF 123	116941971-116941984	GACCAGCAGGGGGT
mCTCF 124	116948219-116948232	GACCAGCAGGGGGC
mCTCF 125	116987985-116987998	GACCAGCAGATGGC
mCTCF 126	116997456-116997469	GACCAGCAGGGGGC
mCTCF 127	117009581-117009594	GACCAGCAGGGGGT
mCTCF 128	117017849-117017862	GACCAGCTGGGGGC
mCTCF 129	117042097-117042110	GACCAGCAGGGGGC
mCTCF 130	117051131-117051144	GACCAGCAGGGGGT
mCTCF 131	117059039-117059052	GACCAGCAGGGGGT
mCTCF 132	117076109-117076122	GACCAGCAGATGGC
mCTCF 133	117090109-117090122	GACCAGCAGATGGC
mCTCF 134	117103500-117103513	GACCAGCAGATGGC
mCTCF 135	117110868-117110881	GACCAGCAGGGGTC
mCTCF 136	117129081-117129094	GACCAGCAGGGGTC
mCTCF 137	117153848-117153861	GACCAGCAGATGGC
mCTCF 138	117162415-117162428	GACCAGCAGGGGTC
mCTCF 139	117189326-117189339	GACCAGCAAGAGGC
mCTCF 140	117193905-117193918	GACCAGCAGATGGC
mCTCF 141	117205930-117205943	GACCAGCAGGGGTC
mCTCF 142	117241369-117241382	GAGCAGCAGGGGTC
mCTCF 143	117249267-117249280	AACCAGCAGGGGGC
mCTCF 144	117287906-117287919	GACCATCAGGGAGC

Table S1. Murine IgH locus contains 144 putative CTCF binding sites. Computationally-predicted CTCF binding sites within the murine IgH locus are labeled mCTCF.1 through mCTCF.144. Sites that are associated with a VH gene segment are shown in black (with the associated VH gene segment listed in the fourth column), while those that are not are shown in red. The sequence of the predicted CTCF binding site is shown, with the central 5 G's within the core sequence shown in blue, while mismatches from the mVH-CTCF consensus sequence are highlighted in yellow. The distance (in base-pairs) from the end of the RSS to the start of the putative CTCF binding site is shown in the last column.

Human Immunoglobulin Heavy Chain Locus

CTCF Site	Aligned Sequence	Associated VH Segment
hCTCF.188	AACCACTAGGGGGC	IGHVIII-82pg
hCTCF.187	GACAACCAGGGGGT	
hCTCF.186	AGCCACCAGGGGGC	
hCTCF.185	TGCCACCAGGGGGA	
hCTCF.184	GACCAGCAGGGGGC	IGHV4-80pg
hCTCF.183	CACCAGCAGGGGGC	
hCTCF.182	AACCACCAAGGGGA	IGHVIII-76-1pg
hCTCF.181	AGCCACCAGGGGGC	
hCTCF.180	AACCACCTGGGGGT	
hCTCF.179	GGACACCAGGGGGC	
hCTCF.178	AACCACCAGGGGGC	
hCTCF.177	CACCACCAGGGGGC	
hCTCF.176	AACCACCTGGGGGT	
hCTCF.175	AACCACCAGGGGGC	
hCTCF.174	AACCACCAGGGGGC	
hCTCF.173	AACCAACAGGGGGC	
hCTCF.172	GAACACCAAGGGGC	
hCTCF.171	GACCACGAGGGGGT	IGHV3-74
hCTCF.170	GGCTACCAGGGGGC	IGHV3-73
hCTCF.169	GGCCAACAGGGGGC	IGHV3-72
hCTCF.168	GACCACCAGGGGGC	IGHV3-71pg
hCTCF.167	GACCACCTGGGGGC	
hCTCF.166	GACCACCAGGGGTC	
hCTCF.165	AAGCACCAGGGGGC	IGHVIII-67-4pg
hCTCF.164	CAACACAAGGGGGC	
hCTCF.163	ATCCACCAGGGGGC	
hCTCF.162	GACCACGAGGGGAC	
hCTCF.161	GGACACCAGGGGGC	
hCTCF.160	GATCACCAGGGGGC	
hCTCF.159	GGACACCAGGGGGC	
hCTCF.158	AACCACCTGGGGGT	
hCTCF.157	GACCTGCAGGGGGC	
hCTCF.156	AACCACCAGGGGGC	
hCTCF.155	GACCACTAGGGGGC	
hCTCF.154	GACCAGCAGGGGGT	
hCTCF.153	AACCACCAGGGGGC	
hCTCF.152	AACCTGCAGGGGGC	
hCTCF.151	AACCACCAGGTGGC	
hCTCF.150	GACCACCAGGGGGC	
hCTCF.149	AACCACCAGGGGGC	
hCTCF.148	GACCACCAGGAGGC	
hCTCF.147	GGACACCAGGGGGC	
hCTCF.146	GGACACCAGGGGGC	
hCTCF.145	GGACACCAGGGGGC	

hCTCF.144	AACCACCAGCGGGC	
hCTCF.143	GGACACCAGGGGGC	
hCTCF.142	AACCACCAGTGGGC	
hCTCF.141	GGACACCAGGGGGC	
hCTCF.140	AACCACCAGCGGGC	
hCTCF.139	GGACACCAGGGGGC	
hCTCF.138	AACCACCAGCGGGC	
hCTCF.137	AACCACCAGGGGGC	
hCTCF.136	GACCAGCAGGTGGC	IGHV4-61
hCTCF.135	CGCCAGCAGGGGGC	IGHV4-59
hCTCF.134	AACCACCAGGGGGC	
hCTCF.133	TACCACGAGGGGGC	
hCTCF.132	AACCACCAGGGGGG	
hCTCF.131	GACCAACAGGGGGC	IGHV3-55pg
hCTCF.130	AGCCACAAGGGGGC	IGHV3-52pg
hCTCF.129	GGACACCAGGGGGC	IGHVIII-49-1pg
hCTCF.128	AGCCACCAGGGGGC	IGHV3-49
hCTCF.127	GACCACCAGGGGGC	IGHV3-48
hCTCF.126	AACCACCAGGGGGT	IGHVIII-47-1pg
hCTCF.125	AACCACCAGGTGGT	
hCTCF.124	AACCACCAGGAAGC	
hCTCF.123	AACCACCAGGGGGC	
hCTCF.122	GAACACCAGAGGGC	
hCTCF.121	AACCACCAGGGGGC	
hCTCF.120	GACCACCAGGGGTC	
hCTCF.119	GACCACCTGGGGGC	
hCTCF.118	CACCCCCAGGTGGC	
hCTCF.117	CACCTCCAGGCGGC	
hCTCF.116	GGCCAGCAGGGGGC	IGHV4-39
hCTCF.115	AACCACCAGGGGGC	IGHVIII-38-1pg
hCTCF.114	AATCACCAGGGGGC	
hCTCF.113	AACCACCAAGGGGC	
hCTCF.112	AACCACCAGGGGGC	
hCTCF.111	AACCACTAGGGGGC	
hCTCF.110	AACCATCAGGGGGC	
hCTCF.109	AACCACCAGGGGGC	
hCTCF.108	AACCACCAGGGGGT	
hCTCF.107	GAACACCAGGGGGC	
hCTCF.106	AACCACCAGGGAGC	
hCTCF.105	GACCAGCAGGTGGC	IGHV4-34
hCTCF.104	GACCAGCAGGGGGC	IGHV4-31
hCTCF.103	GACGAGCAGGGGGC	IGHV4-28
hCTCF.102	AACCACCAGGGAGC	
hCTCF.101	AACCACCATGGGGT	IGHVIII-26-1pg
hCTCF.100	AACCACTAGGGGAC	
hCTCF.99	AACCACCAGGGGGC	
hCTCF.98	AACCACCAGAGGGC	
hCTCF.97	AACCACCAGGTGGC	
hCTCF.96	CACCACCAGGGGGC	
hCTCF.95	GGACACCAGGGGGC	
hCTCF.94	AACCAACAGGGGGC	
hCTCF.93	AACCACAAGGGGGC	

hCTCF.92	GGTCACCAGGGGGC	
hCTCF.91	AACCACCAGGGGAC	
hCTCF.90	GACCACCCGGGGGC	
hCTCF.89	GACCACCACGGGGC	
hCTCF.88	AACCACCAGGGTGT	IGHVIII-16-1pg
hCTCF.87	GATCACGAGGGGGC	
hCTCF.86	AATCACAAGGGGGC	
hCTCF.85	GACCACCAGGGGGC	
hCTCF.84	GGACACCAGGGGGC	
hCTCF.83	AACCACCAGGGGGC	
hCTCF.82	TACCACGAGGGGGC	
hCTCF.81	AACCACCAGGGGGC	
hCTCF.80	GGCCACCAGGGGGC	IGHV3-15
hCTCF.79	AACCACCAGGGGGT	IGHVIII-13-1pg
hCTCF.78	AACCACCAGGGGGC	
hCTCF.77	AACCACCAGGGGGC	
hCTCF.76	AACCACCAGGGGGC	
hCTCF.75	AACCACCAGGGGGC	
hCTCF.74	AACCACCAGGGGGC	
hCTCF.73	AACCACCAGGGGAC	
hCTCF.72	AACCACCAGGGGGC	
hCTCF.71	AACCACCAGGAGGT	
hCTCF.70	AACCACTAGGGGGA	
hCTCF.69	GGACACCAGGGGGC	
hCTCF.68	AACCACCAGTGGGC	
hCTCF.67	GACCTACAGGGGGC	
hCTCF.66	GACCACCAGGGGGC	IGHV3-11
hCTCF.65	GACCACCTGGGGGC	
hCTCF.64	GACCACCAGGGGGC	IGHV3-7
hCTCF.63	GACCAGCAGGGGGC	
hCTCF.62	GGACACCAGGGGGC	
hCTCF.61	GACCAGCAGGGGGC	IGHV4-4
hCTCF.60	AACCACCAGGGAGC	
hCTCF.59	AACCACCAGGGGGC	IGHVIII-2-1pg
hCTCF.58	AACCACCAGGAGGG	
hCTCF.57	GGACACCAGGGGGC	
hCTCF.56	AACCACCAGGGGGC	
hCTCF.55	AACCACCAGGAGGC	
hCTCF.54	GGACACCAGGGGGC	
hCTCF.53	AATCACTAGGGGGC	
hCTCF.52	AACCGCCAGGGGGC	
hCTCF.51	AACCACCAGGGGGC	
hCTCF.50	AACCACCAGGAGGG	
hCTCF.49	GGACACCAGGGGGC	
hCTCF.48	GACCACAAGGGGGC	
hCTCF.47	AACCACCAGGGGGC	
hCTCF.46	AACCACCAGGAGGG	
hCTCF.45	GACCACCAGGAGGT	
hCTCF.44	GGACACCAGGGGGC	
hCTCF.43	AATCACTAGGGGGC	
hCTCF.42	AACCGCCAGGGGGC	
hCTCF.41	AACCACCAGGAGGC	

hCTCF.40	AACCACCAGGAGGT
hCTCF.39	GGACACCAGGGGGC
hCTCF.38	AATCACTAGGGGGC
hCTCF.37	AACCGCCAGGGGGC
hCTCF.36	AACCACCAGGGGGC
hCTCF.35	AACCACCAGGAGGG
hCTCF.34	GGACACCAGGGGGC
hCTCF.33	GACCACAAGGGGGC
hCTCF.32	AACCACCAGGGGGC
hCTCF.31	AACCACCAGGAGGG
hCTCF.30	GACCACCAGGAGGT
hCTCF.29	GGACACCAGGGGGC
hCTCF.28	AATCACTAGGGGGC
hCTCF.27	AACCGCCAGGGGGC
hCTCF.26	AACCGCCAAGGGGC
hCTCF.25	AAGCACCAGGGGGC
hCTCF.24	AACCACCAGGGGAT
hCTCF.23	AACCACCAGGGGGC
hCTCF.22	AACCACCAGGGGGC
hCTCF.21	AACCACCAGGGGAC
hCTCF.20	AACCACCAGGGGAT
hCTCF.19	AACCACCAGGGGTC
hCTCF.18	AACCACCAGGGGGC
hCTCF.17	AACCACCAGGGGGC
hCTCF.16	AACCACCAGGGGGC
hCTCF.15	AACCACCAGGGGGC
hCTCF.14	AACCACCAGGGGGG
hCTCF.13	AACCACCAGGGGGC
hCTCF.12	AACCACCAGGGGGG
hCTCF.11	AACCACCAGGGGGC
hCTCF.10	AACCACCAGTGGGT
hCTCF.9	CACCACCAGAGGGC
hCTCF.8	CACCACCAGGGGGC
hCTCF.7	CACCACCAGGGGGG
hCTCF.6	GACCACCAGGCGGC
hCTCF.5	GACCAGCAGGGGGC
hCTCF.4	CACCACCAGGGCGC
hCTCF.3	CCCCACCAGGGGGC
hCTCF.2	CACCACCAGGGCGC
hCTCF.1	CCCCACCAGGGGGC

Table S2. Human IgH locus contains 188 putative CTCF binding sites. Computationally-predicted CTCF binding sites within the human IgH locus are labeled hCTCF.1 through hCTCF.188. Sites that are associated with a VH gene segment are shown in black (with the associated VH gene segment listed in the third column), while those that are not are shown in red. Sites that are clustered together are boxed in black.

	Size (Mb)	V_H CTCF Sites	Sites / 100kb
Mouse IgH	2.76	140	5.07
Mouse Igκ	3.18	8	0.25
Mouse Igλ	0.23	1	0.43
Mouse TCRβ	0.70	1	0.14
Mouse TCRαδ	1.67	21	1.26
Mouse TCRγ	0.17	0	0.00
Mouse Genome	2,624.08	20,736	0.79
Human IgH	1.28	188	14.69
Human Igκ	0.92	10	1.09
Human Igλ	0.91	15	1.65
Human TCRβ	0.68	1	0.15
Human TCRαδ	1.07	5	0.47
Human TCRγ	0.14	0	0.00
Human Genome	3,076.23	9,752	0.32
Chimpanzee IgH	1.28	118	9.22
Rabbit IgH	0.60	25	4.17

Table S3. The prevalence of VH CTCF sites in the IgH domain is evolutionarily conserved. The density of CTCF binding sites per 100 kb is shown for each of the murine antigen receptor loci, each of the human antigen receptor loci, the chimpanzee IgH locus, and the rabbit IgH locus.

EMSA Probe	Primer A	Primer B
KL1	gaaactctcctgtgaatcca	cattgtgtgtcttgcacagt
KL2	gtctgaggtctgaggacaca	gggttgaatattttgaaggt
KL3	gcctgtaaaattgtctccaa	attggtgtttctcaggggtt
mCTCF.5	actgtgcaagacacacaatgagca	ggctgtaactctgtatttcacaactctg
mCTCF.57	ccacctgcaacttcagttgtaaaccc	agttccctccacagccaccttat
mCTCF.140	ccagagaatgcaacttccgttgaagc	gggcacagtgtctcttccatatcca
mCTCF.108	agccagacaatgcaacttcag	ccctacatatccttctcacctctc
mCTCF.79	tgctgaagtcacactgggcaacta	cagagtaatcccatacccacatctc
mCTCF.65	tggcactgggcagctatagtgtaa	ccacagaggatacagagtaatccc
mCTCF.139	tgggcctcttgctggtctttcttt	gccatctcacaatcacaactctgac
mCTCF.45	gttcacaaccttcagatttgtact	tgtccacgtcagtcatgagcaa
J558.69.170	ggactctgcggtctattact	tacaaagctgacaaaggcac
hCTCF.169	aggacacggccgtgtattac	agagtcctgcaaaaacaaac

Enhancer Blocking Fragment	Primer A	Primer B
7183.2.3	gaaggcgcgccacaaaaggaaactaaattaa	gaaggcgcgccatttatttacatctgatatg
J558.69.170	gaaggcgcgccatgtgttaacattgtcccat	gaaggcgcgccaaactacattatttaacagt
IGHV3-72	gaaggcgcgccatattctgcaaatatgtaaa	gaaggcgcgccttggctattttaaaatgacc

Taqman Probe		Primer A	Primer B
mCTCF.113	ccctgctggtccagaactcacctgc	accttacctgcccttagactttatg	atctctctgctgactatgcttgg
mCTCF.92	cctgctggtcctgtgcatcctcaaagg	ggagatttgaagacacatagaacagg	ctctcttagtgaaagttgaatctacacc
mCTCF.58	tgcaggagattcttggctccattttgccc	ctttactgctgaagtcacactgg	agatacagagtcctccacaacatc
mCTCF.57	tgctccacacccaccccacctgc	aaccctccgttttgcctctatg	gaggattgttggtctcattgtgc
mCTCF.15	ccctgctggtcctggatgctctgc	tgaggacacagccatgtattactg	atcagaactccatatgctctctgc
mCTCF.5	accagacaccggcccaactgagac	aacggagcagaaactgaagaaatg	gcatccctgaactgagtcctatg
Ig3'RR-HS7	tgtcacactagctggttgcctaagca	aatctggtgtctggcactgaggaa	actacgctgtggtcattgtggaga
J558.16.106	tccgcagcctgacatctgaggaca	cctccagcacagcctacatg	ctcaggatgtgtttgtagcactg
JH3	tggccccagtaagcaaaccaggca	gagccctagccaaggatcatttat	tgcagagacagtgaccagagt
MTA1	ttccgtactacctgcttcagcacca	agccagaaacctaagtcagccgtt	ttcaccgtaccttgtcctgtcctt
IL-5	tggctggctctcattcacactgcaagg	gtgccatgactgtgcctctg	agttcagttacacggagaagtaagg

Table S4. PCR primers and Taqman probes used in this study. In the PCR primers used to clone the enhancer blocking fragments, the AscI site is underlined.

ChIP-chip	Start	End	In-silico match
mPeak_1	114,815,940	114,818,094	mCTCF_5
mPeak_2	114,825,632	114,827,388	mCTCF_6
mPeak_3	114,828,004	114,828,430	
mPeak_4	114,834,454	114,836,964	mCTCF_7
mPeak_5	114,848,384	114,851,304	mCTCF_8
mPeak_6	114,861,888	114,865,048	mCTCF_9
mPeak_7	114,891,120	114,892,260	mCTCF_10
mPeak_8	114,899,096	114,901,262	mCTCF_11
mPeak_9	114,922,900	114,924,577	mCTCF_12
mPeak_10	114,925,216	114,925,646	
mPeak_11	114,938,546	114,941,716	mCTCF_13
mPeak_12	114,952,658	114,956,156	mCTCF_14
mPeak_13	114,962,524	114,962,534	
mPeak_14	114,972,464	114,975,605	mCTCF_15
mPeak_15	115,000,376	115,001,324	mCTCF_16
mPeak_16	115,007,810	115,009,102	mCTCF_17
mPeak_17	115,033,670	115,034,980	mCTCF_18
mPeak_18	115,064,162	115,065,574	mCTCF_20
mPeak_19	115,096,451	115,096,900	
mPeak_20	115,097,070	115,098,266	mCTCF_22
mPeak_21	115,115,850	115,116,624	
mPeak_22	115,116,992	115,117,894	mCTCF_23
mPeak_23	115,134,370	115,135,212	mCTCF_24
mPeak_24	115,150,149	115,150,484	mCTCF_25
mPeak_25	115,185,701	115,187,468	mCTCF_26
mPeak_26	115,202,290	115,202,840	mCTCF_27
mPeak_27	115,218,376	115,221,332	mCTCF_29
mPeak_28	115,232,602	115,233,236	
mPeak_29	115,249,164	115,249,401	
mPeak_30	115,250,375	115,252,988	mCTCF_30
mPeak_31	115,271,350	115,272,746	mCTCF_31
mPeak_32	115,285,374	115,287,839	mCTCF_32
mPeak_33	115,298,028	115,298,614	
mPeak_34	115,301,466	115,301,997	
mPeak_35	115,372,672	115,374,129	
mPeak_36	115,391,066	115,392,118	mCTCF_36
mPeak_37	115,414,479	115,415,258	
mPeak_38	115,436,780	115,439,066	
mPeak_39	115,460,887	115,461,474	mCTCF_38
mPeak_40	115,500,756	115,501,274	mCTCF_40
mPeak_41	115,504,478	115,504,518	

mPeak_42	115,526,266	115,526,753	mCTCF_41
mPeak_43	115,529,079	115,529,836	mCTCF_42
mPeak_44	115,560,004	115,561,054	mCTCF_44
mPeak_45	115,562,076	115,562,804	
mPeak_46	115,569,998	115,571,663	
mPeak_47	115,595,797	115,596,013	mCTCF_46
mPeak_48	115,604,217	115,605,672	mCTCF_47
mPeak_49	115,616,192	115,616,262	
mPeak_50	115,629,786	115,629,941	mCTCF_48
mPeak_51	115,644,426	115,645,118	
mPeak_52	115,672,144	115,673,694	
mPeak_53	115,692,764	115,693,878	
mPeak_54	115,698,568	115,699,342	
mPeak_55	115,710,174	115,711,705	mCTCF_49
mPeak_56	115,716,796	115,717,862	mCTCF_50
mPeak_57	115,728,656	115,730,256	
mPeak_58	115,732,310	115,732,472	
mPeak_59	115,733,684	115,734,752	mCTCF_51
mPeak_60	115,754,710	115,755,932	mCTCF_52
mPeak_61	115,780,020	115,781,606	mCTCF_54
mPeak_62	115,785,308	115,785,864	mCTCF_55
mPeak_63	115,821,700	115,822,235	
mPeak_64	115,824,680	115,827,256	mCTCF_57
mPeak_65	115,850,480	115,851,289	
mPeak_66	115,852,490	115,854,674	mCTCF_58
mPeak_67	115,888,286	115,888,344	
mPeak_68	115,888,582	115,889,056	mCTCF_60
mPeak_69	115,906,379	115,908,074	mCTCF_61
mPeak_70	115,922,948	115,924,224	mCTCF_63
mPeak_71	115,949,120	115,950,041	mCTCF_64
mPeak_72	115,964,098	115,965,071	
mPeak_73	115,980,890	115,981,326	mCTCF_65
mPeak_74	115,986,700	115,987,818	mCTCF_66
mPeak_75	115,993,673	115,994,372	mCTCF_67
mPeak_76	116,013,460	116,015,130	mCTCF_68
mPeak_77	116,023,560	116,023,750	
mPeak_78	116,028,882	116,029,906	mCTCF_70
mPeak_79	116,057,532	116,058,629	mCTCF_71
mPeak_80	116,084,056	116,084,610	mCTCF_74
mPeak_81	116,094,356	116,095,378	mCTCF_75
mPeak_82	116,107,809	116,108,346	
mPeak_83	116,120,246	116,121,260	mCTCF_76
mPeak_84	116,136,602	116,137,764	mCTCF_77

mPeak_85	116,165,818	116,166,462	mCTCF_79
mPeak_86	116,166,796	116,167,468	
mPeak_87	116,177,448	116,178,645	mCTCF_80
mPeak_88	116,179,160	116,179,194	
mPeak_89	116,219,374	116,220,826	mCTCF_83
mPeak_90	116,234,864	116,235,874	mCTCF_84
mPeak_91	116,246,345	116,248,354	mCTCF_85
mPeak_92	116,257,946	116,259,771	
mPeak_93	116,305,812	116,306,381	
mPeak_94	116,309,370	116,310,496	mCTCF_87
mPeak_95	116,355,986	116,356,370	
mPeak_96	116,357,732	116,358,884	
mPeak_97	116,359,524	116,360,451	mCTCF_89
mPeak_98	116,383,800	116,385,720	mCTCF_90
mPeak_99	116,387,544	116,387,696	
mPeak_100	116,396,343	116,399,697	mCTCF_91
mPeak_101	116,404,040	116,404,640	
mPeak_102	116,408,806	116,411,646	mCTCF_92
mPeak_103	116,419,226	116,419,594	mCTCF_93
mPeak_104	116,431,826	116,432,460	
mPeak_105	116,435,511	116,436,232	
mPeak_106	116,446,242	116,449,382	mCTCF_94
mPeak_107	116,460,345	116,462,212	mCTCF_96
mPeak_108	116,468,174	116,468,814	mCTCF_97
mPeak_109	116,483,360	116,483,381	
mPeak_110	116,484,752	116,486,046	
mPeak_111	116,499,594	116,501,752	mCTCF_98
mPeak_112	116,515,052	116,515,634	mCTCF_99
mPeak_113	116,532,288	116,532,841	
mPeak_114	116,535,882	116,536,985	mCTCF_100
mPeak_115	116,540,449	116,540,553	
mPeak_116	116,572,968	116,574,180	
mPeak_117	116,574,886	116,575,749	mCTCF_102
mPeak_118	116,597,466	116,599,362	mCTCF_103
mPeak_119	116,601,202	116,601,285	
mPeak_120	116,610,100	116,613,304	mCTCF_104
mPeak_121	116,628,286	116,629,574	mCTCF_105
mPeak_122	116,645,318	116,646,507	mCTCF_106
mPeak_123	116,652,285	116,652,778	mCTCF_107
mPeak_124	116,684,294	116,684,998	
mPeak_125	116,686,071	116,686,966	
mPeak_126	116,690,650	116,692,050	mCTCF_108
mPeak_127	116,698,928	116,698,940	

mPeak_128	116,699,110	116,702,294	mCTCF_109
mPeak_129	116,706,694	116,707,140	
mPeak_130	116,711,512	116,713,498	mCTCF_110
mPeak_131	116,713,834	116,713,952	
mPeak_132	116,714,652	116,714,676	
mPeak_133	116,721,297	116,721,798	
mPeak_134	116,733,700	116,734,456	
mPeak_135	116,736,978	116,738,328	mCTCF_112
mPeak_136	116,745,487	116,748,804	mCTCF_113
mPeak_137	116,762,034	116,763,743	mCTCF_114
mPeak_138	116,770,970	116,772,212	mCTCF_115
mPeak_139	116,786,294	116,786,803	
mPeak_140	116,805,378	116,805,982	
mPeak_141	116,812,292	116,813,486	mCTCF_116
mPeak_142	116,831,114	116,831,760	
mPeak_143	116,832,936	116,836,265	mCTCF_117
mPeak_144	116,841,959	116,844,684	mCTCF_118
mPeak_145	116,853,834	116,853,844	
mPeak_146	116,859,408	116,859,992	
mPeak_147	116,860,918	116,863,488	mCTCF_120
mPeak_148	116,874,148	116,875,692	
mPeak_149	116,876,420	116,876,530	
mPeak_150	116,876,774	116,877,020	
mPeak_151	116,886,021	116,886,803	
mPeak_152	116,893,362	116,895,025	mCTCF_121
mPeak_153	116,902,434	116,903,272	mCTCF_122
mPeak_154	116,929,964	116,930,660	
mPeak_155	116,931,852	116,932,420	
mPeak_156	116,933,517	116,934,676	
mPeak_157	116,941,464	116,942,600	mCTCF_123
mPeak_158	116,947,928	116,948,808	mCTCF_124
mPeak_159	116,980,320	116,980,804	
mPeak_160	116,982,266	116,982,870	
mPeak_161	116,987,456	116,988,908	mCTCF_125
mPeak_162	116,995,950	116,999,306	mCTCF_126
mPeak_163	117,003,636	117,004,126	
mPeak_164	117,008,276	117,011,418	mCTCF_127
mPeak_165	117,017,812	117,018,074	mCTCF_128
mPeak_166	117,028,637	117,029,406	
mPeak_167	117,032,444	117,033,240	
mPeak_168	117,040,763	117,043,927	mCTCF_129
mPeak_169	117,050,564	117,051,924	mCTCF_130
mPeak_170	117,058,496	117,059,886	mCTCF_131

mPeak_171	117,060,774	117,061,000	
mPeak_172	117,072,042	117,072,468	
mPeak_173	117,073,680	117,074,594	
mPeak_174	117,075,238	117,077,084	mCTCF_132
mPeak_175	117,087,792	117,088,644	
mPeak_176	117,089,362	117,091,102	mCTCF_133
mPeak_177	117,100,338	117,100,404	
mPeak_178	117,103,388	117,104,427	mCTCF_134
mPeak_179	117,110,442	117,111,138	mCTCF_135
mPeak_180	117,153,692	117,154,379	mCTCF_137
mPeak_181	117,161,972	117,162,682	mCTCF_138
mPeak_182	117,192,519	117,192,918	
mPeak_183	117,193,788	117,194,192	mCTCF_140
mPeak_184	117,205,482	117,206,200	mCTCF_141
mPeak_185	117,218,797	117,219,212	
mPeak_186	117,230,641	117,231,162	
mPeak_187	117,233,330	117,233,776	
mPeak_188	117,234,700	117,235,934	
mPeak_189	117,241,075	117,241,498	mCTCF_142
mPeak_190	117,248,058	117,250,382	mCTCF_143

Table S5. CTCF sites identified by ChIP-chip largely overlap with those predicted computationally. CTCF sites within the murine IgH locus that were identified by ChIP-chip are labeled mPeak_1 through mPeak_190. Computationally-predicted CTCF binding sites with the murine IgH locus are labeled mCTCF_1 through mCTCF_144.
