# Supplementary material for: The murine IgH locus contains a distinct DNA sequence motif for the chromatin regulatory factor CTCF
Source: J Biol Chem. 2019 Jul 8;294(37):13580–92. doi: 10.1074/jbc.RA118.007348 (PMC6746451; doi:10.1074/jbc.RA118.007348)
Supplement: Supporting Information [file supp_RA118.007348_Supplementary_Methods.pdf]

Supplementary Methods

Cell culture

CD19$^+$ cells were plated at 5×10^6 cells/ml in 10 cm plates previously layered with OP9 stromal cells and grown in IMDM medium containing 2% heat inactivated fetal calf serum, 0.03% Primatone, 200 U/mL penicillin, 200 U/mL streptomycin, 4 mM L-glutamine, and 50 μM β-mercaptoethanol supplemented with the indicated amount of recombinant IL-7 (R&D Systems) for 3 days prior to transfer to a new feeder layer of Mitomycin C (Sigma) treated ST2 stromal cells and allowed to grow for an additional day prior to harvest. CD19$^+$ bone marrow cells and cultured pro B cells were purified with ficoll-paque (GE Healthcare Life Science) immediately prior to harvesting for chromatin. The number of live CD19$^+$ B220$^+$ cells was determined by FACS. Cells were stained with the following antibodies from BioLegend: PE/Cy5 anti-mouse CD19; FITC anti-mouse B220; PE/Cy5 Rat IgG2a, κ Isotype control; and FITC Rat IgG2b, κ Isotype control. Cell sorting and analysis were performed on FACSCalibur (Becton Dickinson) using CellQuest Pro software and the data analyzed by FlowJo.

Sequence Alignments

Annotated genomic sequence spanning antigen receptor loci was obtained from Genbank via the following accession numbers: murine: IgH locus (BN000872); Igκ NG_005612; Igλ (NG_004051); TCRβ (AE000663-65); murine TCRα/δ (AE008683-86; TCRγ (AF021335 & AF037352); human: IgH (NG_001019); Igκ (NG_000833); Igλ (NG_000002); TCRβ (NG_001333); TCRα/δ (NG_001332); TCRγ (NG_001336; rabbit IgH (AY386694-98; partial).

Electrophoretic Mobility Shift Assays

DNA binding reactions with IVT CTCF contained IVT Buffer (10mM Tris-Cl pH7.5, 10mM NaCl, 10mM KCl, 10mM $MgCl_2$, 0.1mM $ZnSO_4$, 0.5mM EDTA, 10% glycerol), 2ng end-labeled probe (see above), 2μg poly-dI:dC (USB) non-specific competitor DNA, and 1μL IVT protein in a 20μL reaction volume. DNA binding reactions with nuclear extracts contained Nuclear Extract Buffer (20mM HEPES pH 7.9, 60mM KCl, 0.1mM $ZnSO_4$, 1mM EDTA, 1mM DTT, 12% glycerol), 2ng end-labeled probe, 2μg poly-dI:dC, and 3-5μg nuclear extract in a 20μL reaction volume. Binding reactions were incubated at 25°C for 30 min. and resolved on a 4% acrylamide, 0.5X TBE gel. For supershift assays, 1μL of anti-CTCF rabbit polyclonal IgG (Upstate Biotechnologies) or 1μL of anti-Set9 rabbit polyclonal IgG (Upstate Biotechnologies) was added to the reaction after the initial 30 min. incubation and binding was allowed to proceed for an additional 20 min at 25°C.

Binding assays with purified CTCF were carried out in 10 mM Tris (pH 7.5), 10 mM NaCl, 5 mM $MgCl_2$, 10 mM KCl, 0.1 mM $ZnSO_4$, 0.5 mM EDTA, and 10% glycerol. Each 20μl reaction contained 2 ng of ^{32}P-labeled substrate DNA, 10 ng of CTCF, and 2 μg of poly(dI-dC) competitor. All binding reactions were incubated at 25°C for 30 minutes. Reaction products were separated through 4% polyacrylamide-TBE gels (19:1 acrylamide-bisacrylamide, 22.5 mM Tris, 22.5 mM boric acid, 0.5 mM EDTA). Gels were visualized by autoradiography.

Enhancer Blocking Assay

Approximately $1x10^7$ K562 cells were transfected by electroporation with 0.5pmol linearized *pNI*-based test constructs and 0.5pmol linearized *pHyg*^{R} (a gift from Brian Seed).

After 2-3 weeks of selection in soft agar, the number of G418- and hygromycin-resistant colonies was counted. To account for varying degrees of transfection efficiency, the number of G418-resistant colonies was normalized to the number of hygromycin-resistant colonies within each transfection. This value was normalized to the number of G418-resistant colonies (independently normalized to hygromycin-resistant colonies) obtained from a transfection with *pNI*.

The control plasmids, *pJC3-4* and *pJC13-1*, as well as the backbone plasmid, *pNI*, were generously provided by Gary Felsenfeld (NIH). A 2.3kb XbaI fragment containing the λ phage DNA was excised from pJC3-4 and blunt-cloned into the AscI site of *pNI* to create *pNI+λDNA*. A 1.2kb KpnI fragment containing the chicken β-globin 5'HS4 insulator (INS) was isolated from pJC13-1 and blunt-cloned into the AscI site of *pNI* to create *pNI+INS*. A tandem repeat of the INS fragment was obtained by partial XbaI digestion of pJC13-1 and the resulting 2.4kb fragment was blunt-cloned into the AscI site of *pNI* to create *pNI+2xINS*. A 1.425kb DNA fragment spanning the entire 7183.2.3 coding sequence was PCR amplified from Pro B cell genomic DNA with primers containing AscI linkers (See Table S4) and either cloned with TOPO TA into *pCR2.1-TOPO* creating *p7183.2.3-TOPO* or digested with AscI and cloned into the AscI site of *pNI* to create *pNI+mCTCF.5*.

DNA manipulations for enhancer-blocking assay

pNI+2x(mCTCF.5) was created by partially digesting *pNI+mCTCF.5* with AscI, followed by the insertion of an additional mCTCF.5 fragment. A 2.85kb DNA fragment was recovered from an AscI partial digest of *pNI+2x(mCTCF.5)* and blunt-cloned into the NdeI site of *pNI* to create *pNI+2x(mCTCF.5)@NdeI*. PCR-based mutagenesis was used to construct

pNI+2x(mCTCF.5)^{mut}. Linearized *p7183.2.3-TOPO* DNA was used as a template for PCR with the following primers: 5'-TGCAGAGCATCC AGGACCAGGCAGTTTGCGCGGAGAGCA-3' (upper) and 5'-TTAGTTGACAATATTGGTGTTTCTCAGGGG-3' (lower). An aliquot of this reaction was then used as the template for a second round of PCR using the same lower primer combined with the following upper primer: 5'-GAGCTCAAACTAAAACCTCCTGCAGAGCATCCAGG-3'. The resulting product was TOPO TA cloned into *pCR2.1-TOPO* and subsequently digested with SacI and HincII. This mutant 343bp SacI-HincII fragment was exchanged for the wildtype SacI-HincII fragment in *p7183.2.3-TOPO*. The 1.425kb *mCTCF.5*^{mut} was isolated by AscI digestion, cloned into the AscI site of *pNI*, and double inserts were created as described above. 1.425kb DNA fragments spanning the entire J558.69.170 and IGHV3-72 coding sequences were PCR amplified from Pro B cell genomic DNA and human HeLa cell DNA, respectively, with primers containing AscI linkers (See Table S4). After gel extraction and purification, the fragments were digested with AscI, cloned into the AscI site of *pNI*, and double inserts were constructed as described previously to create *pNI+2x*V_H*J558* and *pNI+2x(hCTCF.169)*. A 2.4kb DNA fragment containing two copies of the chicken β-globin 5'HS4 INS was isolated from a partial XbaI digest of *pJC13-1*. and cloned into an XbaI digested *pNI* backbone creating *pNI-2xINS*. *pNI-2xINS+2x(mCTCF.5)* and *pNI-2xINS+2x(mCTCF.5*^{mut}) were created in *pNI-2xINS* as described above.

Chromatin Immunoprecipitation

Chromatin immunoprecipitations were performed as described with 30 μl of anti-CTCF antibody (Upstate Biotechnology). Antibody-bound DNA was collected by absorption to

Protein A resin (Sigma). Real-time PCR was carried out using 1 ng of DNA at 95°C for 10 min, followed by 50 cycles of 94°C for 30 sec, 52°C for 30 sec, and 60°C for 1 min, and 60°C for 1 min. For real-time PCR with TaqMan® probes, triplicate reactions containing 1 ng of IP or input sample, 20 pmol of each primer, and 125 nM specific dual-labeled fluorogenic probe (IDT) were amplified in TaqMan® Universal PCR Master Mix (Applied Biosystems). Fold-enrichment values were calculated as R^$(Ct_{Input}–Ct_{IP})$; where R represents the rate of amplification, Ct represents the cycle threshold value, and both values represent an average of each triplicate set of reactions.
